# Epitope-focused immunogens targeting the hepatitis C virus glycoproteins induce broadly neutralizing antibodies

**DOI:** 10.1126/sciadv.ado2600

**Published:** 2024-12-06

**Authors:** Kumar Nagarathinam, Andreas Scheck, Maurice Labuhn, Luisa J. Ströh, Elisabeth Herold, Barbora Veselkova, Sarah Tune, Johannes T. Cramer, Stéphane Rosset, Sabrina S. Vollers, Dorothea Bankwitz, Matthias Ballmaier, Heike Böning, Edith Roth, Tanvi Khera, Henrike P. Ahsendorf-Abidi, Oliver Dittrich-Breiholz, Jonas Obleser, Michael Nassal, Hans-Martin Jäck, Thomas Pietschmann, Bruno E. Correia, Thomas Krey

**Affiliations:** ^1^ Institute of Biochemistry, Center of Structural and Cell Biology in Medicine, University of Lübeck, 23562 Lübeck, Germany.; ^2^ Institute of Bioengineering, École Polytechnique Fédérale de Lausanne, Lausanne CH-1015, Switzerland.; ^3^ Swiss Institute of Bioinformatics (SIB), Lausanne CH-1015, Switzerland.; ^4^ Institute for Experimental Virology, TWINCORE, Centre for Experimental and Clinical Infection Research, a joint venture between the Helmholtz Centre for Infection Research and the Hannover Medical School, 30625 Hannover, Germany.; ^5^ Institute of Virology, Hannover Medical School, 30625 Hannover, Germany.; ^6^ Department of Psychology, University of Lübeck, 23562 Lübeck, Germany.; ^7^ Center of Brain, Behavior, and Metabolism, University of Lübeck, 23562 Lübeck, Germany.; ^8^ Central Research Facility Cell Sorting, Hannover Medical School, 30625 Hannover, Germany.; ^9^ Division of Molecular Immunology, Department of Internal Medicine 3, Friedrich-Alexander University of Erlangen-Nürnberg, 91054 Erlangen, Germany.; ^10^ Research Core Unit Genomics, Hannover Medical School, 30625 Hannover, Germany.; ^11^ Department of Internal Medicine 2/Molecular Biology, University Hospital Freiburg, 79106 Freiburg, Germany.; ^12^ German Center for Infection Research (DZIF), partner site Hannover-Braunschweig, 30625 Hannover, Germany.; ^13^ Excellence Cluster 2155 RESIST, Hannover Medical School, 30625 Hannover, Germany.; ^14^ German Center for Infection Research (DZIF), partner site Hamburg-Lübeck-Borstel-Riems, 38124 Braunschweig, Germany.; ^15^ Centre for Structural Systems Biology (CSSB), 22607 Hamburg, Germany.

## Abstract

Hepatitis C virus (HCV) infection causes ~290,000 annual human deaths despite the highly effective antiviral treatment available. Several viral immune evasion mechanisms have hampered the development of an effective vaccine against HCV, among them the remarkable conformational flexibility within neutralization epitopes in the HCV antigens. Here, we report the design of epitope-focused immunogens displaying two distinct HCV cross-neutralization epitopes. We show that these immunogens induce a pronounced, broadly neutralizing antibody response in laboratory and transgenic human antibody mice. Monoclonal human antibodies isolated from immunized human antibody mice specifically recognized the grafted epitopes and neutralized four diverse HCV strains. Our results highlight a promising strategy for developing HCV immunogens and provide an encouraging paradigm for targeting structurally flexible epitopes to improve the induction of neutralizing antibodies.

## Teaser

Structure-based HCV immunogens elicit an epitope-focused immune response cross-neutralizing multiple HCV genotypes.

## INTRODUCTION

An estimated 58 million people worldwide are chronically infected with the hepatitis C virus (HCV), resulting in 290,000 annual deaths, primarily due to liver cirrhosis and hepatocellular carcinoma (*1*). Although the approved direct-acting antivirals reach impressive cure rates for patients with chronic hepatitis C (*2*), it is apparent that WHO’s goal to eliminate HCV as a major public health threat by 2030 will not be achieved. This mainly results from the lack of awareness of patients with HCV caused by the asymptomatic nature of acute and early chronic phase of HCV infection together with the absence of nationwide screening programs, as this combination renders the implementation of comprehensive treatment strategies difficult. Consequently, HCV incidence remains high with approximately 1.5 million infections occurring annually. Modeling studies predict this global epidemic to continue in the absence of a prophylactic vaccine (*3*).

Accumulating evidence suggests that both B and T cell immunity are important for the control of an acute HCV infection [reviewed in (*4*, *5*)], suggesting that an effective prophylactic HCV vaccine likely requires an immunogen that robustly elicits broadly neutralizing antibodies (bnAbs) in combination with an efficient T cell response (*6*). The two HCV glycoproteins E1 and E2 form heterodimers, and this E1E2 glycoprotein complex interacts with cellular receptor molecules including the scavenger receptor class B type 1 (*7*) and the tetraspanin CD81 (*8*, *9*) during virus entry. This interaction activates signaling pathways that promote HCV-CD81 movement to tight-junction regions, where interactions with claudin 1 and occludin support viral internalization (*10*, *11*).

To date, only two prophylactic vaccine candidates have progressed to clinical trials in humans but with limited success. An adenoviral vector–based T cell vaccine candidate encoding the nonstructural proteins of a single HCV isolate failed to protect against chronic HCV infection in a cohort of people who inject drugs at high risk of virus transmission (*12*). Although a B cell vaccine candidate comprising MF-59–adjuvanted viral glycoprotein E1/E2 heterodimers protected chimpanzees from challenge with HCV of the same genotype, in a phase 1 trial, bnAbs were only induced in a small fraction of individuals (*13*, *14*). In general, HCV vaccine development has been hampered by the huge genetic and antigenic diversity of HCV, with eight genotypes differing in their nucleotide sequence by up to 30% that frequently results in evasion from both B and T cell responses [reviewed in (*4*, *5*)]. Additional evasion mechanisms include, among others, decoy epitopes in the glycoproteins, extensive glycosylation, and a remarkable structural flexibility within conserved neutralization epitopes.

These glycoprotein-related features likely hamper the capacity of recombinant HCV glycoproteins to induce bnAbs, as observed both in humans and mice (*14*, *15*). The wealth of available structural data together with recent progress in protein design methodology offers a strategy to overcome these limitations by transplanting important neutralization epitopes onto preexisting or de novo protein scaffolds (*16*). Such an epitope-focused immunogen will present the grafted neutralization epitope on the surface of a multimeric nanoparticle (NP) and shape the induced antibody response (*17*). This strategy has been successfully applied to several important viral pathogens such as HIV-1, respiratory syncytial virus, and Ebola virus (*17*–*19*). While structure-based immunogen design was previously applied to HCV (*20*–*23*), the designed immunogens either did not induce a potent nAb response, were not evaluated in vivo, or were based on engineering of a soluble E2 ectodomain.

The major determinants for the induction of a humoral immune response against HCV are located on the glycoproteins E1 and E2 (*24*–*32*). They include the composite CD81-binding site and the metastable E2 domain presenting antigenic region 4 [AR4, (*33*)], which are targeted by potent bnAbs. These two regions and their interactions with bnAbs have been structurally characterized in detail (*9*, *34*–*42*). Together with biophysical studies including hydrogen deuterium exchange combined with mass spectrometry (*43*, *44*), these structural data reveal that, in particular, the composite CD81-binding site adopts multiple conformations—an intrinsic structural flexibility that likely hampers the induction of bnAbs [reviewed in (*44*, *45*)]. Epitope I (residues 412 to 423) is a prime example of such a structural flexibility, as epitope peptides comprising this segment have been crystallized in complex with nAb fragments in three distinct conformations (*37*, *40*, *46*). This suggests that the conformation of this amino acid segment follows an “induced-fit” binding mode to the antibody, and this conformational flexibility could explain the limited immunogenicity to the epitope I observed in patients with HCV (*47*).

To address these limitations, we generated epitope-focused immunogens based on two antigenic regions including one on HCV E1 (residues 314 to 324) and one on HCV E2 (epitope I). We demonstrate that a cocktail of these immunogens induced a robust antibody response both in laboratory mice and mice with a human antibody immunoglobulin G (IgG) locus [human antibody mice, (*48*)]. These antibodies neutralized a diverse panel of HCV strains in cell culture and monoclonal human antibodies isolated from immunized human antibody mice, closely reflecting a human antibody response, neutralized the same virus panel, and recognized the grafted epitope. Our approach demonstrates that epitope-focused immunogens are a versatile alternative to develop vaccines for cases like HCV where glycoprotein engineering remains challenging. Moreover, our NPs represent a promising candidate to develop into an effective B cell vaccine.

## RESULTS

### Computational design of E1 epitope or E2 epitope I immunogens

**Fig. 1.**
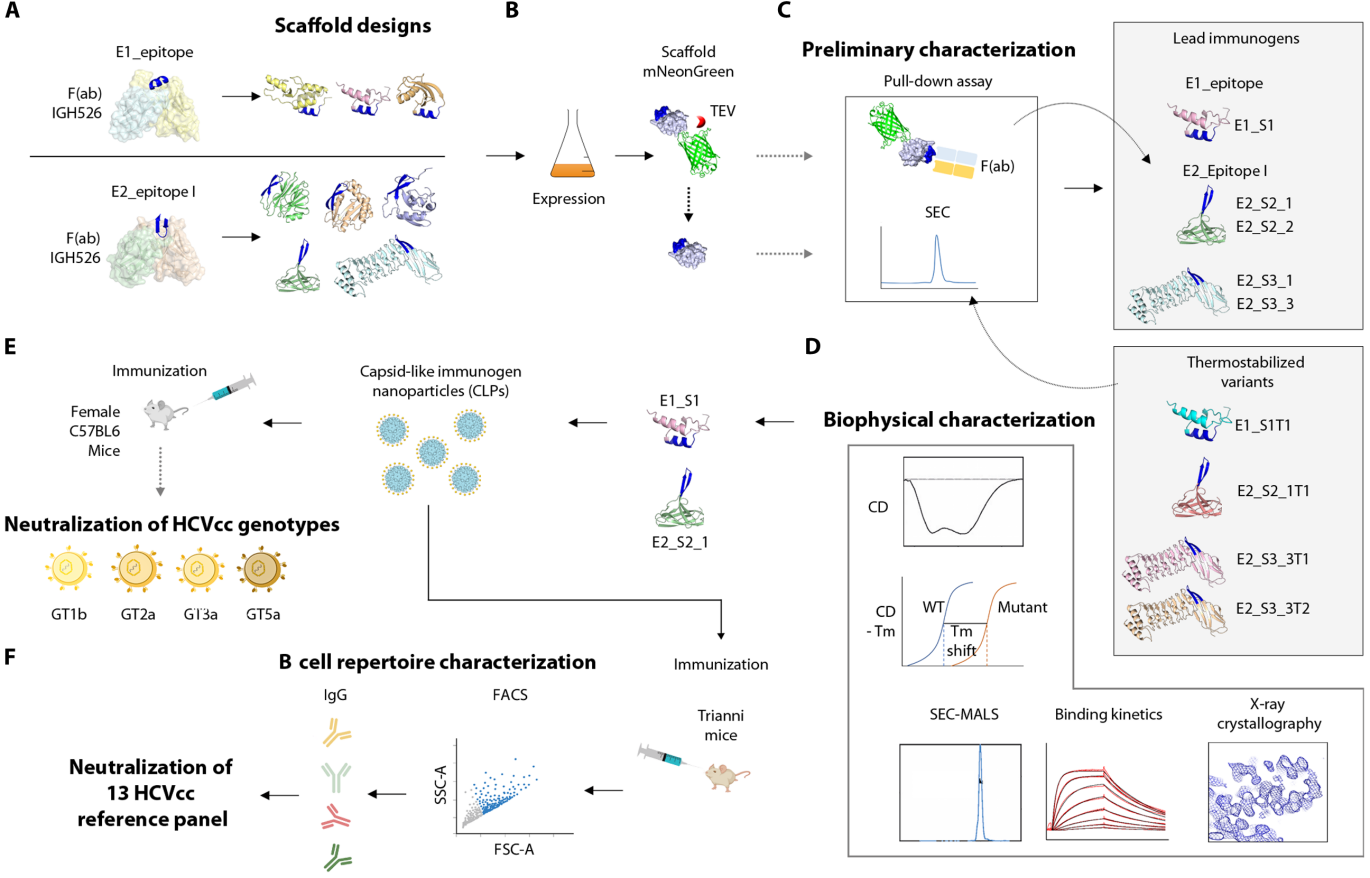
Pipeline for characterization of lead immunogen candidates. (**A**) Epitopes were extracted from crystal structures of the respective epitope within E1 (residues 314 to 324) and E2 (residues 412 to 423, epitope I) in complex with their cognate Fab fragments (PDB 4N0Y and 4DGV). Multiple designs were generated for each of the epitopes by grafting their side chains onto suitable scaffolds followed by in silico structure–based design to optimize epitope display and antibody recognition. Side chains of epitopes were transplanted onto suitable scaffolds followed by further structure-based design to optimize epitope display and antibody recognition. (**B** and **C**) Fusion with the fluorophore mNeonGreen allowed rapid identification of soluble monomeric scaffolds that expose an accessible epitope by pull-down assay. Promising candidates were subjected to more extensive computational design to increase protein stability (i.e., thermostabilization). (**D**) Thermostabilized designs together with the parental scaffolds were subjected to biochemical and structural characterization, resulting in the identification of promising lead immunogens. (**E**) The latter were displayed on hepatitis B virus core antigen capsid-like particles to augment immunogenicity and used for immunization of mice. Neutralization potency of mouse antisera was evaluated against a representative panel of HCVcc. (**F**) Antigen-specific B cells were isolated from mice encoding a fully human IgG locus (human antibody mice) that were immunized with the immunogen NPs and monoclonal antibodies were isolated that broadly neutralize a more extensive reference panel comprising HCVcc of 13 distinct HCV strains (*56*) and specifically bind to the two displayed target epitopes.

One possible approach to overcome the structural flexibility is structure-based, epitope-focused vaccine design, which has recently been successfully used to design immunogens mimicking epitopes of important human pathogens (*16*–*19*). Two HCV neutralization epitopes, one within E1 [E1_Epi; amino acids 314 to 324; Protein Data Bank (PDB) 4N0Y] adopting an α-helical conformation (*38*) and epitope I within E2 (E2_EpiI; amino acids 413 to 423; PDB 4DGV) in β-hairpin conformation (*28*, *41*, *49*–*52*) were selected as epitopes of interest because they comprise linear amino acid segments encoding defined secondary structure elements, suggesting high amenability to computational immunogen design procedures. The crystal structures of the two epitopes were used as templates to identify scaffolds closely matching the backbone conformation among a database of 238,276 monomers from the PDB (fig. S1). Initial bioinformatic analysis together with a visual inspection of the superposition onto the complex identified three unique scaffolds for E1_Epi (PDB 2HFQ, 1Z6U, and 1W4K) and five for E2_EpiI (PDB 1KRI, 2EXN, 2K6Z, 4CIL, and 2CY2; Fig. 1 and fig. S1).

Twenty-three individual variants derived from these unique scaffolds were further characterized with respect to antibody binding and their oligomerization state. Pull-down assays with the respective Fab fragments together with size exclusion chromatography (SEC) identified three promising immunogen candidates, including designs 1W4K_08 (targeting E1_Epi, hereafter referred to as E1_S1), 2K6Z_01, and 4CIL_03 (both targeting E2_EpiI, hereafter referred to as E2_S2_1 and E2_S3_3, respectively) (fig. S2 and table S2). All three designs were subjected to in silico thermostabilization (see Materials and Methods) to further improve their physicochemical properties. In this manner, one to three in silico thermostabilized variants (denoted as “T”) were generated from each of the parental scaffolds. Thermostabilized designs alongside the parental scaffolds were biophysically characterized allowing for identification of one lead immunogen candidate for each epitope (Fig. 1).

**Fig. 2.**
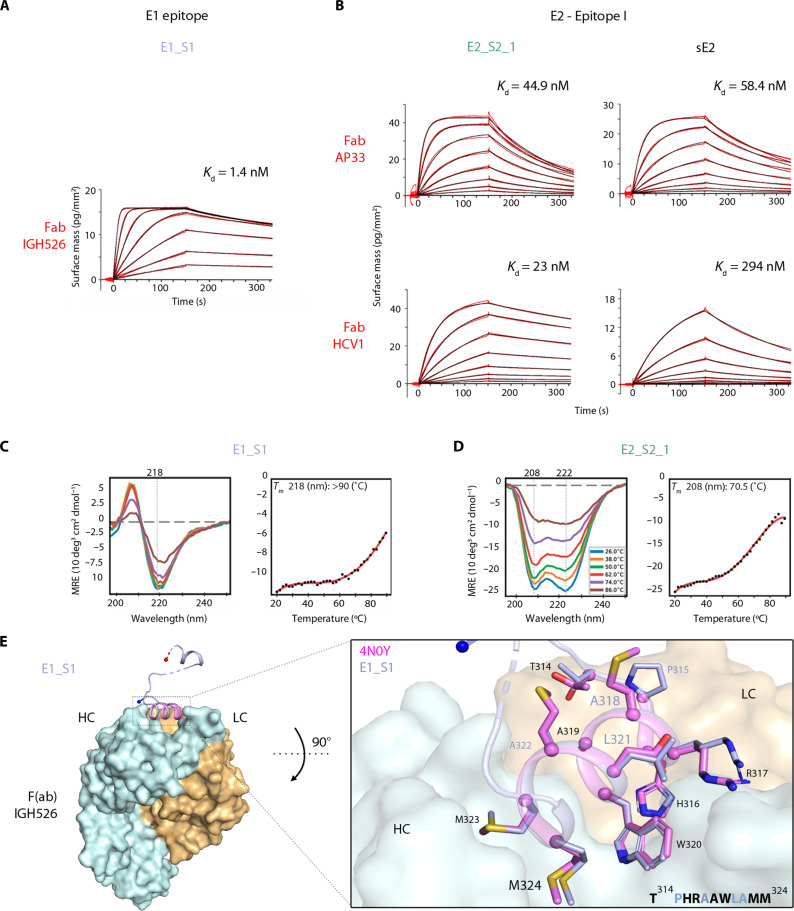
Characterization of lead immunogen candidates for NP production. (**A**) Grating-coupled interferometry (GCI)–derived kinetic binding parameters for Fab (ligand)–scaffold (analyte) interactions. Sensorgrams are shown with data in red and their respective fitting in black. The dissociation constants *K*_d_ for a 1-to-1 binding model or a mass transport binding model of the immunogen candidates–cognate Fab interactions are shown). Binding kinetics of E2_EpiI scaffolds E2_S2_1 and E2_S3_3 against Fabs AP33 and HCV1 using sE2 as control are shown. (**B**) Similarly, binding kinetics was also assessed for E1_S1 scaffold against Fab IGH526. (**C** and **D**) CD spectra reported as mean residual ellipticity (MRE) were measured for E1_S1 (C) and E2_S2_1 (D) at different temperatures. The data points extracted from negative maxima from each of the CD spectra are indicated as black dots and fitted with a curve in red; the apparent CD-*T*_m_ is derived from the slope. Typical positive maxima or negative minima of proteins with α-helical or β strand secondary structures are indicated with gray dotted lines. (**E**) Crystal structure of scaffold E1_S1 in complex with heavy chain (HC) and light chain (LC) of the IGH526 Fab fragment. (Right) A close-up view of the grafted epitope from the scaffold is overlaid with the reported epitope structure (PDB 4N0Y). Epitope residue side chains of the scaffold complex structure (purple) or the reported PDB model (pink) are highlighted as sticks, Cα atoms as spheres. The sequence of the grafted epitope in the scaffold highlights the mutated residues in purple (E1_S1).

The secondary structure and thermal stability of individual designs was analyzed by circular dichroism (CD) spectroscopy. Both thermostabilized variants of E1_S1 displayed a typical α-helical spectrum in line with the reported crystal structure of the parental scaffold and a melting temperature (*T*_m_) of ~70°C (Fig. 2 and fig. S3). In contrast, the CD spectrum of E2_S2_1, E2_S2_2, and E2_S2_1T1 variants suggested a β strand–dominated secondary structure in agreement with the reported structure and a *T*_m_ of >90°C. Of note, design E2_S2_1 exhibited a superior expression level when compared with other E2_S2–derived designs (Fig. 2 and fig. S3). The CD spectra of E2_S3_1, E2_S3_3, E2_S3_3T1, and E2_S3_3T2 variants indicated a mixed α/β secondary structure and showed a lower *T*_m_ compared to the three E2_S2 variants (fig. S3). SEC combined with multiangle light scattering (MALS) revealed a single oligomeric species for each of the designs in agreement with a theoretical molecular weight of ~6.5 kDa (E1_S1), 14.2 kDa (E2_S2), and 32.8 kDa (E2_S3; fig. S4). In summary, secondary structure and thermal stability analysis revealed all three lead immunogens to be monomeric proteins with an overall fold resembling their parental scaffold.

To determine the kinetic binding parameters of individual immunogens, we carried out surface plasmon resonance analysis of the interaction between individual designs and the respective Fabs. E1_S1 and Fab IGH526 as well as E2_S2_1 and both Fab AP33 and Fab HCV1 (Fig. 2, fig. S2, and table S5) interacted with nanomolar affinity. Comparative analysis of the kinetic binding parameters of the latter interactions with the interaction between the two Fabs and a soluble HCV E2 ectodomain (sE2) revealed similar binding kinetics, underscoring that the computational design process succeeded in mimicking the interface accurately. In contrast, design E2_S3_3 bound only to Fab HCV1, but not to Fab AP33, suggesting different binding requirements for both Fabs to this scaffold (figs. S2 and S5).

Crystallographic analysis of E1_S1 in complex with the Fab IGH526 revealed unambiguous electron density only for parts of the four design molecules within the asymmetric unit. Density was only observed in two complexes for the N-terminal α helix comprising the epitope. In contrast, in two complexes, density for both the N- and C-terminal α helices was visible, albeit lacking the linker region connecting them. While these results suggest that stability of the scaffold can be improved, this conformational flexibility within the scaffold did apparently not affect binding to Fab IGH526. This was further demonstrated by the conformation of the 2.5-turn α helix carrying the epitope, which is identical in all four individual complexes and resembles the structure of the peptide in complex with the Fab (PDB 4N0Y; Fig. 2) (*38*).

The crystal structure of E2_S3_3T1 in the absence of Fab (fig. S5) reveals a conformation closely resembling the designed model, with the β-hairpin epitope located in the Ig-like inter-repeat domain. Superposition of AP33 and HCV1 Fab complexes onto the designed E2_S3_3T1 structure revealed steric hindrance of Fab AP33 but not Fab HCV1 with juxtaposed loops. The steric clashes are caused by a variation in the angle of approach of the two Fabs to the epitope, which differs by 22° between HCV1 and AP33 (*51*). Together with the low thermostability profile of E2_S3 variants, a scaffold size that might hinder the assembly of multivalent NPs for immunization and the apparent limited antibody binding of E2_S3 variants, these results identified E1_S1 and E2_S2_1 as the most promising immunogen candidates.

### Characterization of multivalent scaffold immunogen NPs

**Fig. 3.**
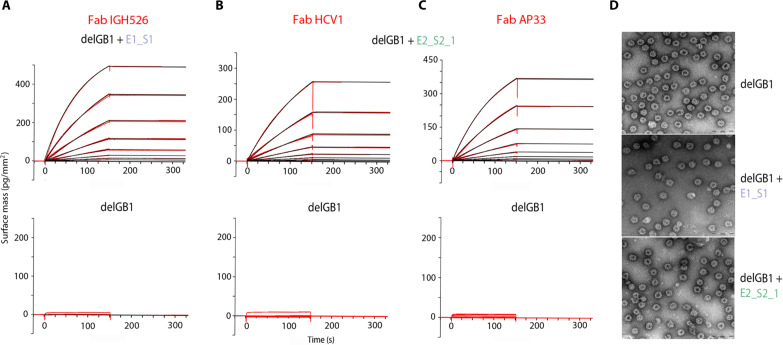
Characterization of capsid-like immunogen NPs. (**A** to **C**) GCI-derived binding kinetics for Fab-NP interactions. Sensorgrams are shown with data in red and their respective fits in black. Kinetic parameters for a 1-to-1 binding model or a mass transport binding model did not yield any productive values because of the absence of observable dissociation due to the avidity effect caused by the multivalent nature of the NPs. (**D**) Negative stain electron microscopy of NPs presenting either scaffold lacking GB1 (delGB1). Scale bar, 200 nm.

Multimerization of immunogens in form of regular NPs is known to improve their immunogenicity via improved B cell receptor clustering, antigen presentation, and T follicular helper cell engagement (*53*). Multimerization of the relatively hydrophobic HCV epitopes was attempted on several self-assembling protein NP platforms (e.g., Ferritin, Lumazine synthetase, Ficolin-2, or SpyCatcher-Spy Tag system) but did not give rise to immunogen NPs, likely due to aggregation induced by the molecular crowding of scaffold on the NP surface. We therefore further optimized the hepatitis B virus core antigen NP platform to allow for scaffold display at lower occupancy on the NP surface (fig. S6) (*54*). The chosen coexpression approach making use of the solubility enhancer GB1 allowed production of soluble NPs presenting E1_S1 or E2_S2_1, respectively, with an occupancy of ~25 to 38% on the NP surface (fig. S7). GB1 was proteolytically removed from icosahedral T-3 or T-4 capsids (delGB1), and kinetic binding parameters of these particles to the respective Fab fragment revealed almost no dissociation, indicating a high-affinity interaction due to a strong avidity effect caused by the multivalent nature of the scaffold NPs (Fig. 3, A to C). The NP integrity was assessed by negative-stain electron microscopy, indicating an average diameter of ≤50 nm (Fig. 3).

### Induction of bnAbs by epitope-focused immunogens in mice

To evaluate the capacity of the scaffold NPs to induce glycoprotein-reactive antibodies, we immunized mice following different protocols involving scaffold NPs and a soluble E2 ectodomain [the patient-derived genotype 2a glycoprotein 2a-3; sE2; (*55*)]. Seventeen mice were immunized with either scaffold NPs [equivalent to a total of ~9 μg scaffold; E1_S1_HBc (hepatitis B core) (NP E1 Epi), E2_S2_1_HBc (NP E2 EpiI), or a combination of both NP populations (NPmix)], a prime-boost strategy (3X with sE2, boost with E2_S2_1_HBc), sE2 alone, or HBc_GFP (green fluorescent protein) particles lacking scaffold and GB1 as control.

Serum enzyme-linked immunosorbent assay (ELISA) analysis revealed that the two groups immunized with sE2 alone or in combination with E2_EpiI as a prime-boost strategy developed a strong antibody response targeting a UKN2b_2.8 sE2. In contrast, scaffold NPs induced very low levels of antibodies binding to sE2 at the standard serum dilution used in this ELISA (fig. S8A), which became more apparent in a lower serum dilution, indicating that very low antibody levels were induced by scaffold NPs (fig. S8B).

**Fig. 4.**
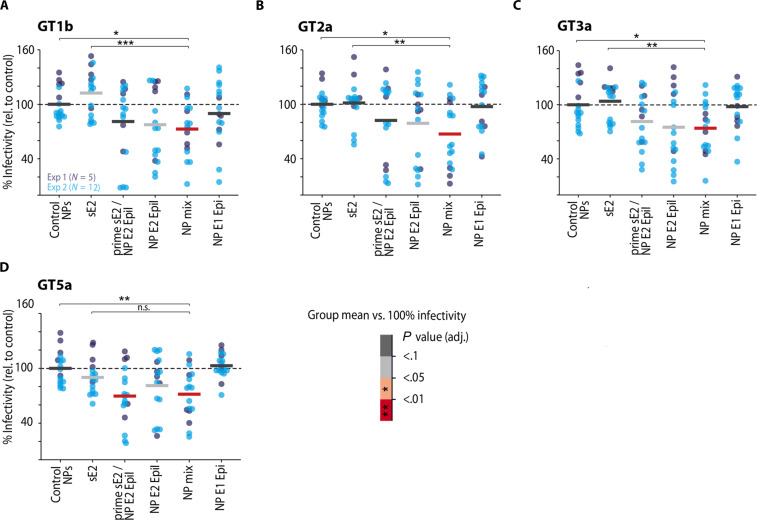
NP mix induces bnAbs in C57BL6 mice. A single-dose neutralization assay was used to investigate the induction of nAbs against a panel of four different viruses that differ in neutralization sensitivity (Gt1b_J4, Gt2a_Jc1, Gt3a_S52, and Gt5a_SA13). (**A** to **D**). Female C57BL6 mice were immunized with the indicated immunogen(s) in two batches [N1 = 5 (dark blue), N2 = 12 (light blue)], and the neutralization efficiency of the individual mouse sera is reported as % infectivity (defined as the % infectivity normalized to mice immunized with control NPs). Colored horizontal bars indicate the arithmetic mean per immunization protocol. Warm colors indicate immunization protocols with a significant mean change in % infectivity (two-sided one-sample *t* test against a value of 100% infectivity, false discovery rate–corrected for multiple comparisons). Neutralization efficiency was analyzed with linear mixed-effects regression indicating that inter-mouse variability accounted for 54% of total variance (intra-class correlation coefficient, ICC = 0.54). Neutralization of NPmix was compared to sE2 and control by general linear tests with Dunnett-correction for multiple comparisons. To satisfy linear model assumption, raw % infectivity values were Box-Cox transformed (λ = 1.4) for statistical analysis. n.s., *P* > 0.05; *P ≤ 0.05; **P ≤ 0.01; ***P ≤ 0.001.

To quantify the fraction of nAbs within immune sera, we performed a single-dose neutralization assay using cell culture–derived HCV particles (HCVcc) from a panel of four different HCV isolates (Gt1b_J4, Gt2a_Jc1, Gt3a_S52, and Gt5a_SA13) that differ in genotype and their degree of neutralization resistance (*56*). We quantified neutralization efficiency as individual % infectivity normalized to mice immunized with sE2. A statistical analysis of the proportion of total variance due to inter-mouse variability as opposed to variability due to the immunogen revealed that ~54% of the total observed variability in % infectivity was accounted for by this inter-animal variability (tables S3 and S4) (*57*). Despite this considerable noise level and the low levels of sE2-reactive antibodies described above, we observed a significant cross-neutralizing activity for postimmune sera of mice immunized with epitope-focused immunogens normalized against control NPs (Fig. 4). The induced cross-neutralizing activity was observed with higher statistical power when alternatively analyzed as neutralization ratio (together with the animal-specific preimmune sera; fig. S9). On the other hand, immunization with sE2 did not induce a considerable nAb titer (Fig. 4) despite high sE2-reactive antibody titers (fig. S8A) and in line with previous reports describing a limited capacity of sE2 to induce nAbs (*15*). Serum neutralization titers for the individual NPs remained modest, with NP E2 EpiI consistently showing better induction of bnAbs. This result was in line with the fact that several monoclonal Abs (mAbs) (e.g., AP33, HCV1, and HC33.8) targeting epitope I are potently nAbs and in a direct comparison nAb AP33 neutralized HCVcc more efficiently than nAb IGH526 targeting the epitope within E1 (*58*). Of note, a single dose of NP E2 EpiI used as boost after sE2 priming was sufficient to considerably improve the nAb response, suggesting that the epitope-focusing effect works in a prime-boost scenario. The highest neutralization was achieved by immunizing with a mix of two NP populations displaying one epitope each—implying that activation of naive B cells with two epitopes results in an additive effect when compared with the individual NP populations as well as the sE2 used in this study (Fig. 4). Antibodies induced by the NP mix cross-neutralize different HCV genotypes including strain J6 (GT2a), which is reported to be highly resistant to neutralization (*56*).

Of note, these results illustrate the minute amounts of E2-reactive antibodies that are required for efficient in vitro neutralization and highlight that most antibodies induced by a soluble glycoprotein bind but do not neutralize HCV in vitro. It will be important to address the physiological relevance of these antibodies in infected patients or vaccinated individuals in the future.

### Stimulation of relevant germline genes in human antibody mice via an NP mix displaying two distinct epitopes

**Fig. 5.**
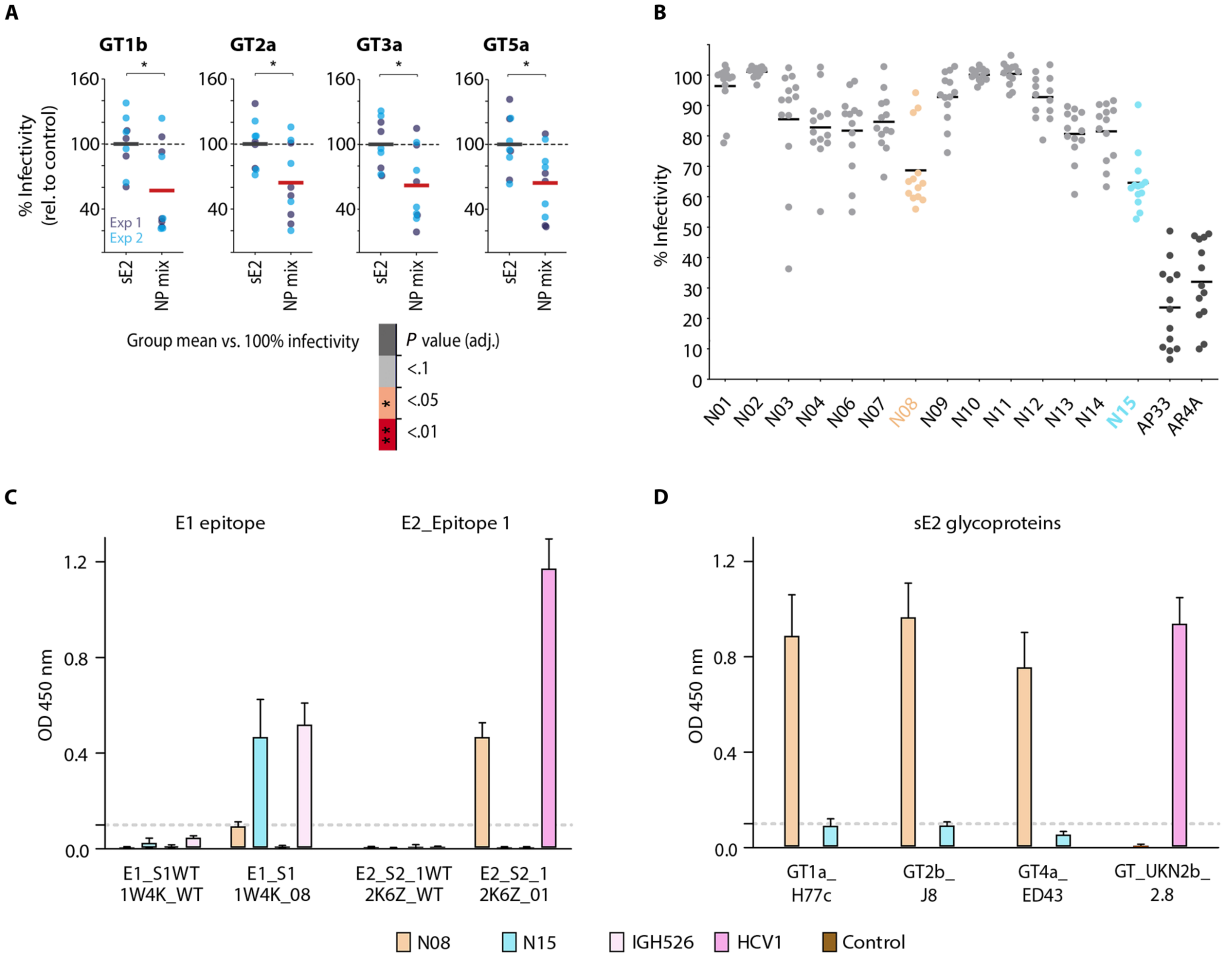
Neutralization of HCVcc genotypes with sera from Human antibody mice and isolated mAbs from B cell sequencing immunized with NPmix or sE2 and ELISA. (**A**) Human antibody mice were immunized with sE2 GT2a_3 or NPmix. Sera from individual mice were evaluated in a single-dose neutralization assay against the panel of viruses described in Fig. 4. (A) Scatterplots show individual % infectivity (normalized to mice immunized with sE2) along with the arithmetic mean per immunization protocol as in Fig. 4. Neutralization efficiency was analyzed with linear mixed-effects regression indicating that inter-mouse variability accounted for 40% of total variance (ICC = 0. 4). Follow-up linear models per genotype compared mean % infectivity of mice immunized with NP mix versus sE2. *P ≤ 0.05 (**B**) A single-dose neutralization assay at 50 μg/ml against a reference panel of 13 HCVcc strains (*56*) was performed with human mAbs isolated from mice immunized with NPmix (N01 to N15) and two well-characterized reference bnAbs (dark gray). Individual % infectivity (normalized to infection in the presence of phosphate-buffered saline) is shown, and each dot represents an independent HCVcc strain mean value (scatterplot) and the median is highlighted. mAbs with the broadest neutralization capacity are colored (N08, orange; N15, cyan). Human bnAbs were further analyzed in ELISA against (**C**) wt (1W4K/2K6Z) or epitope scaffolds E1_S1 (1W4K_08) and E2_S2_1 (2K6Z_01). Parental nAbs IGH526 and HCV1 were used as positive controls and an unrelated human IgG1 antibody as negative control. (**D**) Broad recognition of sE2 via bnAbs N08 and N15 was tested by ELISA using sE2 H77c (GT1a), J8 (GT2b), ED43 (GT4a), and UKN2b_2.8 (GT2b). As sE2 UKN2b_2.8 was not recognized by bnAb N08, the well-characterized bnAb HCV1 was used as positive control in this assay. ELISA results are presented as the mean of three biological replicates with three technical replicates each.

To evaluate the capacity of our NP combination described above to induce a potent nAb response also in the context of a human antibody repertoire, we immunized human antibody mice with NPmix or GT2a-3 sE2. All 44 human variable region of Ig heavy chain (V_H_) regions with all D_H_ and J_H_ gene segments and 39 V_k_ segments with all J_k_ gene segments are used in these animals instead of the cognate mouse genes, thereby creating an antibody repertoire that closely mimics the human antibody repertoire (*48*, *59*). Induction of bnAbs was evaluated by single-dose serum neutralization assay using the same panel of four viruses described above. Sera from animals immunized with NPmix showed a marked reduction in infectivity (~50%) compared to control sera (Fig. 5A), indicating induction of bnAbs also in the context of a human antibody repertoire. Of note, similar to the results observed for C57BL6 mice, a direct ELISA of human antibody mouse sera revealed that scaffold NPs induced very low levels of antibodies binding to sE2 at the standard serum dilution (fig. S8C).

To examine the human antibody response to immunization in more detail, we isolated single HCV-reactive memory B cells from the spleen of immunized human antibody mice by magnetic cell separation and flow cytometric cell sorting (FACS; fig. S10). We obtained 174 productive paired heavy- and light-chain sequences that passed the quality control (fig. S11). Sequence analysis revealed a polyclonal response derived from multiple germlines, with few clonally related sequences (~2 to 7%). Clonotype analysis revealed that VH chains originating from V_H_3-33 were enriched, which is also used by other HCV bnAbs such as HCV1 (fig. S11), suggesting that the designed antigen activates similar germline precursors as the native viral antigen. We selected 15 sequences based on barcode frequency (fig. S11), expressed and purified the corresponding mAbs, and analyzed their neutralization activity via a single-dose neutralization assay against a panel of 13 HCVcc strains (Fig. 5B) (*56*). Two mAbs (N08 and N15) showed broad cross-neutralization capacity, albeit at moderate potency when compared to the well-characterized reference bnAb AP33 and AR4A (*60*) with a cross-neutralization index of 36.6 and 40.7%, respectively.

We analyzed the specificity of both bnAbs in more detail by ELISA against monomeric scaffold proteins E1_S1 and E2_S2_1 as well as the corresponding wt scaffolds lacking the HCV epitope. BnAb N15 recognized E1_S1 but did not interact with the corresponding wt 1W4K protein or with E2_S2_1, whereas bnAb N08 recognized E2_S2_1 but did not interact with the respective wt 2K6Z scaffold or with E1_S1. A comparative sequence analysis revealed that both N08 and N15 drastically differ in heavy chain CDR3 and germline light chain V-gene from the respective parental bnAbs IGH526 and HCV1 used for immunogen design (fig. S12), thereby explaining the observed differences in neutralization potency.

These results were complemented by an ELISA against intact sE2 glycoproteins derived from genotypes ED43 (GT4a), H77c (GT1a), and J8 (GT2b), which were recognized by bnAb N08 targeting epitope I within E2, but not bnAb N15 recognizing an epitope within E1 (Fig. 5). In summary, these data demonstrate that our epitope-focusing immunogens induce epitope-specific bnAbs in the context of a human antibody repertoire and that these antibodies broadly neutralize a representative panel of HCV viruses.

## DISCUSSION

Despite the undisputed medical need for an efficient HCV vaccine that protects against chronic HCV infection, the specific approach to develop such a vaccine remains elusive to date. Several strategies aiming either at an efficient T cell response or a robust nAb response have been tested with limited success (*4*, *61*). This is likely due to the numerous immune evasion mechanisms used by HCV such as its high antigenic diversity, extensive glycosylation and lipid shielding of glycoproteins, and an unprecedented conformational flexibility within the HCV neutralization epitopes (*62*–*64*).

Here, we have demonstrated that small thermally stable protein scaffolds that accurately mimic the structure and specific conformation of two HCV neutralization epitopes in complex with a human bnAb can induce nAbs in the context of a murine and a human antibody repertoire. This study represents a proof of concept that bnAbs targeting conformationally flexible epitopes can be induced by constraining the conformation of the respective epitope in a conformationally more rigid scaffold. Our NP mix induces a bnAb response in the murine and human antibody context.

Overall, the observed neutralization titers can still be improved. However, it is important to note that the obtained results are highly reproducible and significantly exceed the relatively high noise constituted by the high inter-animal variability. The neutralization epitopes targeted by epitope-focusing immunogen design in this study were primarily selected because they are amenable to in silico immunogen design, implying that they might not necessarily be the epitopes that induce the most potent bnAb response. Most anti-HCV plasma neutralizing activity targets known epitopes that are structurally more complex [e.g., the front layer epitope/AR3 (*65*, *66*) or an epitope at the E1/E2 interface/AR4 (*33*, *42*)] and therefore less amenable to the approach used in this study. Recent advances in deep-learning methods to design proteins that contain prespecified functional sites (*67*) might allow designing of immunogens mimicking such complex epitopes. A combination of our NP populations with scaffold NPs displaying additional epitopes is likely to extend the induced bnAb response both in breadth and potency. Eventually, although due to mismatched human leukocyte antigen and antibody genes, Trianni mice might not be ideal for quantifying antibody responses, in a pairwise comparison, the bnAb titers induced in human antibody mice exceeded those induced in C57BL6 mice, suggesting that the efficacy of the NPs to induce bnAbs could be superior in a human antibody context.

Previous vaccination studies suggest that a single-shot vaccination approach will likely be insufficient, and, instead, a prime-boost strategy will be necessary to induce robust cross-nAb responses. In such a setting, glycoprotein-based vaccine candidates—independent of the priming strategy [e.g., mRNA-based approaches (*68*) versus mono- and multivalent protein–based approaches (*69*)]—are likely to also induce non-nAbs. These results highlight that focusing of the polyclonal immune response to specific neutralization epitopes using scaffold NPs will likely improve potency and breadth of the induced antibody response, as suggested in the basic prime-boost protocol reported here. In addition, using epitope-specific immunogen designs displaying distinct epitopes in the analysis of polyclonal sera allows precisely quantifying binding antibodies of different specificities and thereby offers an accurate approach to further analyze the composition of glycoprotein-reactive antibodies in polyclonal sera in more detail. A deconvolution of a polyclonal serum response has already been performed on the basis of neutralization data (*65*) but has not been possible to date based on binding data for glycoprotein-reactive antibodies due to the lack of epitope-specific immunogen designs. Such an analysis would enable a more detailed understanding of the correlates of protection for HCV and thus constitute an important step for rational vaccine design.

In summary, our study shows that structure-based immunogen designs represent a versatile strategy to induce antibodies against conformationally flexible neutralization epitopes in general and a promising alternative for HCV vaccine development. It will be particularly advantageous to create immunogens that focus the antibody responses onto specific HCV neutralization epitopes. Our findings emphasize how such immunogens can be useful in a prime-boost scenario, where the immune system is primed with a glycoprotein-based immunogen and subsequently boosted with the scaffold NPs. In parallel, such epitope-specific scaffolds can also be used as diagnostic tool to qualitatively and quantitatively assess a given polyclonal antibody response and therefore better understand the determinants of efficient neutralization of HCV.

## MATERIALS AND METHODS

### Computational design of epitope-focused immunogens

To identify suitable protein scaffolds, 238,276 monomeric proteins from the PDB (*70*) were queried for protein backbones in agreement with the individual epitopes using the Rosetta macromolecular modeling suite (*71*, *72*). Scaffold candidates were exhaustively analyzed for segments matching the epitope using protein backbone root mean square deviation (RMSD) evaluation.

The E1 epitope (residues 314 to 324) was extracted from the crystal structure of the epitope in complex with nAb IGH526 [PDB ID: 4N0Y; (*38*)], and protein scaffolds with a C_α_-RMSD deviation of ≤0.5 Å were selected if no atomic clashes were present and the predicted binding energy to the corresponding antibody was ~20 to 35 Rosetta energy units (REU). In total, nine unique scaffolds were identified, three of which were selected after visual inspection [PDB IDs: 2HFQ, 1Z6U, and 1W4K; (*73*)].

The E2 epitope I (residues 411 to 423) was extracted from the crystal structure of the epitope-HCV1 antibody complex [PDB ID: 4DGV; (*37*)]. Putative scaffolds were filtered at ≤1.0 Å backbone RMSD, less than five clashing atoms, and a predicted binding affinity of ≤35 REU, resulting in 14 scaffold candidates of which five were retained after visual inspection [PDB IDs: 1KRI (*74*), 2EXN (*75*), 2K6Z (*76*), 4CIL (*77*), 2CY2]. We performed side-chain grafting for each epitope using the MotifGraft protocol (*78*) to transplant the epitope side chains onto the selected scaffolds. Rational design was implemented to resolve steric clashes between epitope residues and scaffold residues, as well as scaffold residues and antibody residues by mutating clashing scaffold positions to appropriate residues, i.e., biochemical properties of wild-type residues were retained if possible. In addition, unnecessary or potentially destabilizing mutations were reverted. Unpaired cysteines were mutated to alanine or serine in all scaffolds based on surface exposure. In designs based on scaffold 2K6Z, the native copper-binding site was removed by introducing mutations at functional residues. Similarly, the catalytic site in scaffold 2CY2 and the zinc-binding site in scaffold 1Z6U were mutated. Furthermore, extraneous domains were trimmed in several scaffolds as well as long loop regions at the N or C terminus that could potentially interfere with antibody binding.

With the described in silico modeling, we arrived at two variants for each of the unique designs (E1_2HFQ and E1_1Z6U) and eight variants for E1_1W4K design all presenting E1 epitope; two variants for each of the four unique designs (E2I_1KRI, E2I_2EXN, E2I_2K6Z, and E2I_2CY2) and three variants for the design E2I_4CIL all carrying E2 epitope I, which were all subjected to in vitro characterization (fig. S1). Promising design candidates from this first round of selection were subjected to additional computational design to increase the thermostability of epitope scaffolds E1_1W4K_08 (E1_S1), E2I_2K6Z_01 (E2_S2_1), and E2I_4CIL_03 (E2_S3_3). We followed a previously described computational procedure (*79*). Briefly, multiple sequence alignments (MSAs) were constructed on the basis of the wild-type amino acid sequence of the epitope scaffolds. The BLASTP algorithm (*80*) was used to collect homologous sequences, and hits were removed if they covered less than 60% of the query sequence or had a sequence identity of less than 30% of the query sequence. In total, a maximum of 500 hits were retrieved at an e-value cutoff of 10^−4^. On the basis of these sequences, an MSA was generated using MUSCLE (*81*), and the resulting MSAs were translated to position-specific scoring matrices (PSSMs) using PSI-BLAST (*82*). To identify potentially stabilizing mutations, we used the generated PSSMs alongside Rosetta design (*83*) allowing all amino acids to mutate except residues within 7 Å of the corresponding antibody, thus not introducing mutations to the binding interfaces of the epitope-focused designs. During the design process, only mutations that scored higher than the current residue type in the PSSM were considered as nonnegative scores are likely to occur during evolution. To keep the number of introduced mutations minimal, we selected mutants only if they improved the overall Rosetta energy of the epitope scaffold by at least 2 REUs.

### Scaffold expression and purification

Codon-optimized computationally designed scaffolds carrying epitopes of HCV were cloned into the pET28a vector by restriction-free cloning. The scaffolds were C-terminally tagged with an mNeonGreen–8× histidine tag connected via a tobacco etch virus (TEV) protease cleavage site and overexpressed in *Escherichia coli* BL21(DE3)-Gold strain. Cells were grown to an optical density at 600 nm (OD_600_) = 0.4 to 1.5 at 220 rpm, 37°C in 1 liter of LB or TB media and induced with 1 mM isopropyl-β-d-thiogalactopyranoside (IPTG) at 18°C for further 16 hours of overexpression. The cells were harvested at 4500*g* with JLA 8.1000 rotor and resuspended in 30 ml of buffer A [50 mM tris (pH 8), 500 mM NaCl, and 40 mM imidazole] containing 1× Roche cOmplete EDTA-free protease inhibitor cocktail, deoxyribonuclease I (DNase I, 10 μg/ml) for 5 g of wet cell pellet. The slurry was lysed using a cell disruptor at 1.5 kbar in two successive rounds, and the lysate was centrifuged at 75,000*g* using a JA25.50 rotor to remove cell debris and membranes. The lysate was filtered through a 0.22-μm filter before subjecting to immobilized nickel affinity chromatography (INAC). HisTrap FF 1 or 5 ml column was washed with 5 CV of buffer A and loaded with the filtered supernatant. The column was washed with 10 to 20 CV of buffer A together with 2% of buffer B to remove unspecifically bound proteins. Further, the scaffolds were eluted in one step with 250 mM imidazole and subjected to gel-filtration chromatography using Superdex 200 Increase 10/300 GL column in GF buffer [10 mM tris (pH 8) and 100 mM NaCl]. Scaffold fusions were cleaved with TEV protease at a ratio of 1:0.25 and mNeonGreen was removed by reverse-INAC to obtain scaffold alone. Cleaved scaffolds were purified by gel filtration chromatography using Superdex 75 10/300 GL in GF buffer. Ten % glycerol was added to all the buffers where required. The proteins as a fusion or apo-form were concentrated to ~5 mg/ml using Amicon 5 or 10 kDa molecular weight cut-off (MWCO) centrifugal filter units. The scaffold fusion or protein alone were spun for 10 min at 18,213*g* and 4°C, and the supernatant was flash-frozen in liquid nitrogen for further analysis or crystallization trials.

### Expression and purification of Fab regions of nAbs

Heavy and light chains of the Fab regions of nAbs IGH526, AP33, and HC84.1 were cloned as codon-optimized synthetic genes into a *Drosophila* S2 Fab expression vector containing two N-terminal BiP-signal sequences and a C-terminal double Strep tag for the heavy chain as reported previously (*84*). For the expression of the HCV1 Fab, synthetic genes for heavy and light chains were cloned into two separate *Drosophila* S2 expression vectors equipped with N-terminal BiP-signal sequences and in the case of the Ig heavy chain with a C-terminal double Strep-tag. The sE2 codon-optimized synthetic gene was cloned into the *Drosophila* S2 vector containing the N-terminal BiP-signal sequences and a C-terminal enterokinase cleavage site followed by a double Strep-tag.

*Drosophila* S2 cells were transfected with the plasmids together with a resistance marker puromycin for selection as described previously (*85*). Stable transfected S2 cell lines were induced at a density of ~6 to 8 × 10^6^ cells/ml for 6 to 7 days with 4 μM CdCl_2_ for large-scale production. Fabs and sE2 were subsequently purified from the supernatant by affinity chromatography Strep Tactin XT Superflow resin (IBA, Göttingen, Germany) in a self-packed column followed by an SEC step (HiLoad 26/600 Superdex 200 pg) in 10 mM tris (pH 8.0) and 100 mM NaCl. The double Strep-tag was proteolytically removed from the sE2 protein using EKmax enterokinase.

### IgGs expression and purification

Synthetic genes of paired heavy and light chain variable regions of antibodies were bought from Twist Biosciences, which were cloned as IgG1 into a pcDNA3.1 expression vector under the control of a cytomegalovirus promoter. Human embryonic kidney ExPi293F cells were transfected using ExpiFectamineTM 293 transfection reagent (both Thermo Fisher Scientific) following the manufacturer’s recommendation with minor modifications. Briefly, a plasmid-DNA cocktail containing also expression-enhancing plasmids encoding the large T antigen of the SV40 virus as well as the cell cycle inhibitors p21 and p27 (p21 + p27 + SV40 at ratios of 0.69:0.05:0.25:0.01, respectively), was prepared in 5 ml of Opti-MEM (Gibco) at a concentration of 1 μg/ml of final culture volume. The ExpiFectamineTM 293 transfection reagent was also diluted in 5 ml of Opti-MEM and incubated for 5 min at room temperature (RT) followed by mixing with the plasmid DNA cocktail, incubation for 20 min at RT, and dropwise addition to the cells. Enhancers were added the next day between 16 and 18 hours, and, after a 5-day expression, the cells were pelleted and IgG was purified by affinity chromatography from the supernatant using a Protein G column (Cytiva) followed by SEC using a Superdex 200 Increase 10/300 GL (Cytiva) equilibrated in 1× phosphate-buffered saline (PBS). Purified mAbs were concentrated and stored at 4°C.

### HBc-immunogen CLPs

For the production of capsid-like particles (CLPs) for immunization, hepatitis B core (HBc) plasmid pRSF-T7-HBc149-GFPopt presenting GB1 protein alone or together with the plasmid pET28a2-HBc149-GFP-H6 carrying scaffold in the position of GB1 were cotransformed into *E. coli* BL21-CodonPlus(DE3)-RIPL strain (*54*). Preculture was inoculated at 0.05 OD_600_ in 1 liter of media with appropriate antibiotics and were grown to 0.6 to 0.8 OD_600_ at 220 rpm, 37°C and induced with 1 mM IPTG at 20°C for further 12 to 16 hours of overexpression. The cells were harvested at 4500*g* using JLA 8.1000 rotor and resuspended in 5 ml of TN300 buffer [25 mM tris-HCl (pH 7.5), 300 mM NaCl, and 1 mM EDTA] for 3 g of cell pellet. Before cell disruption, the resuspended cells were treated with 0.5% Triton X-100, lysozyme (2 mg/ml), and 1× Roche cOmplete EDTA-free protease inhibitor cocktail for 10 to 15 min at 4°C until the solution becomes viscous because of the release of the *E. coli* genomic DNA. Further, the viscous material was incubated for 10 min at RT with DNAse I (0.1 mg/ml) and 5 mM MgCl_2_ and then disrupted at 1.5 kbar in two successive rounds. The lysate was centrifuged at 35000*g* for 30 min using JA25.50 rotor, and the supernatant was filtered through a 0.22-μm filter before sucrose gradient centrifugation. HBc particles presenting both GB1 and the scaffold or GB1 alone were separated by ultracentrifugation using SW32Ti rotor at 28,000 rpm for 3.5 hours layering on a 10 to 60% sucrose gradient bed prepared in TN300 buffer. Fractions of 2 ml were fractionated for SDS–polyacrylamide gel electrophoresis (SDS-PAGE) analysis, and the fractions containing capsid-like immunogen NPs were pooled together and subjected to gel filtration chromatography using HiLoad 26/600 Superdex 200-pg column in capsid buffer [25 mM tris (pH 8), 500 mM NaCl, and 10% glycerol]. TEV protease was used at a ratio of 1:0.25 to cleave GB1 off the CLPs left behind only with scaffold and separated from TEV protease by a second round of gel filtration chromatography as before. Diluted capsid-like immunogen NPs were concentrated by sandwiching 5 g of Aquacide II powder on either side of a 10-cm length 50-kDa MWCO SnakeSkin dialysis tubing filled with 30 ml of NP pool. The concentration was performed at 4°C under constant shaking at 200 rpm until the capsids reached a concentration of 1 mg/ml. Dialysis was performed twice in a 3-kDa MWCO SnakeSkin tubing at 4°C for 4 hours against capsid buffer at a ratio of 1:100 to remove excess of Aquacide II. Alternatively, CLPs were also concentrated using Amicon stirred cell concentrators with a 500-kDa MWCO membrane filters. CLPs were spun for 10 min at 18,213*g*, 4°C to remove aggregates, and the supernatant was flash-frozen in liquid nitrogen for further analysis or immunization.

### Pull-down assay to detect promising immunogens

Multiple computationally designed scaffolds proteins carrying unique epitopes of HCV (E1 epitope and epitope I within E2) were assessed for lead immunogen candidates by Strep-Tactin XT sepharose pull-down assay. nAbs antigen–binding fragments (IGH526, HCV1, and AP33) of the respective HCV epitopes were loaded at quantities of ~100 μg onto to a 0.2-ml bed gravity flow Strep-Tactin sepharose column previously washed with 10 CV of A1 buffer [10 mM tris (pH 8), 150 mM NaCl, and 1 mM EDTA]. Fabs remain bound to the column via C-terminal Twin-Strep-Tag. The column was washed with 10 CV of A1 buffer and loaded with 500 μg of scaffold-mNeonGreen protein. The column was rewashed with 2 CV of A1 buffer and eluted with B1 buffer (A1 buffer + 50 mM biotin). The eluate was analyzed using SDS-PAGE gel electrophoresis followed by Coomassie staining to assess the complex formation of the immunogen with the Fab.

### CD spectroscopy and thermostability measurements

Experiments were performed using a Chirascan V100 CD spectrometer (AppliedPhotonics). Far-ultraviolet CD spectra of designed scaffolds were collected between a wavelength of 190 and 250 nm in a 1-mm path-length quartz cuvette. Proteins were dissolved in PBS buffer (pH 7.4) at concentrations between 20 and 40 μM. Wavelength spectra were averaged from two scans with a scanning speed of 20 nm min^−1^ and a response time of 0.125 s. The thermal denaturation curves were collected by measuring the change in ellipticity at 218 or 222 nm over a temperature range from 20° to 95°C with 2° or 5°C increments. The resulting data were converted to mean residue ellipticity, fitted to a two-state model, and apparent melting temperatures (*T*_m_) were obtained.

### Size exclusion chromatography multiangle laser light scattering

Concentrated proteins were further purified by SEC on a HiLoad 16/600 Superdex 75 pg (GE Healthcare) column in 1× PBS buffer. Protein concentrations were determined by measuring the absorbance at 280 nm on a Nanodrop (Thermo Fisher Scientific) by using the protein’s corresponding extinction coefficient.

MALS was used to assess the monodispersity and molecular weight of the proteins. Samples containing between 50 and 100 μg of protein in PBS buffer (pH 7.4) were injected into a Superdex 75 10/300 GL column (GE Healthcare) using an HPLC system (Ultimate 3000, Thermo Fisher Scientific) at a flow rate of 0.5 ml min^−1^ coupled in-line to an MALS device (miniDAWN TREOS, Wyatt). Static light-scattering signal was recorded from three different scattering angles. The scattering data were analyzed by ASTRA software (version 6.1, Wyatt).

### Crystallization of immunogen-Fab complexes

Purified scaffold and their respective Fab fragments (E1_S1 + Fab IGH526, E2_S3_3T1 alone) were mixed at a ratio of 1:1.5 and incubated overnight at 4°C allowing the formation of scaffold-Fab complex. The scaffold excess was removed by gel filtration chromatography using a HiLoad 26/600 Superdex 200-pg column in GF buffer. The peak fractions were pooled and concentrated to 10 mg/ml for E1_S1–Fab complex and E2_S3_3T1 scaffold alone. All the crystallization trials were performed in SWISSCl MRC 96-well plates. Crystals of the complex and the scaffold alone were grown by the vapor-diffusion sitting drop technique with each drop formed by 100 nl of the precipitant solution and 100 nl of protein equilibrated against 50 μl of the precipitant solution. Crystals of E1_S1–Fab complex grew in the condition, H10 from JCSG+ (Molecular Dimensions) and E2_S3_3T1 crystals grew in the condition, F8 JBScreen Wizard 3 and 4 HTS. Protein crystals were fished from the 96-well sitting drops through 0.5 μl of 20% glycerol cryoprotectant solution layered on top of the crystal drop and directly flash-frozen in liquid nitrogen for x-ray data collection. Data statistics are tabulated in table S1.

### Structure determination and refinement

Diffraction data were integrated by XDS (Kabsch, 1988). The crystals structures were solved using molecular replacement method with phenix.phaser using the appropriate search models; PDB ID: 1W4K, 4CIL for the designed scaffolds and PDB ID: 4N0Y (IGH526) for the Fab region (*86*). Subsequent model building was performed using COOT and further refined using phenix.refine program (*86*). Refinement statistics are provided in table S1. All crystal structure figures were generated with PyMol.

### Grating-coupled interferometry

Experiments were performed with the Creoptix WAVE delta instrument (Creoptix AG) using 4PCH WAVE chips. Fresh 4PCH WAVE chips were conditioned with borate buffer [100 mM sodium borate (pH 9.0) and 1 M NaCl], and then StrepTactin XT (IBA GmbH) was immobilized on the surface to levels of 5600 to 5800 pg/mm^2^ using the standard direct amine coupling approach and a flow rate of 10 μl/min. Briefly, the surface was activated for 7 min with 1:1 (v/v) mix of 365 mM 1-ethyl-3-(3-dimethylaminopropyl)carbodiimide hydrochloride and 100 mM *N*-hydroxysuccinimide (Amine coupling kit, GE Healthcare), followed by the injection of StrepTactinXT (25 μg/ml) in 10 mM sodium acetate (pH 4.5) and finished with a final quenching step with 1 M ethanolamine (pH 8.5) (Amine coupling kit, GE Healthcare) for 7 min.

Multicycle kinetic measurements were performed at 25°C at a flow rate of 120 μl/min with a 1:2 dilution series of the analyte. Therefore, the respective twin strep-tagged Fabs (25 to 200 μg/ml) were captured in every cycle on the surface until the desired density was reached. After the injection of the analyte, the surfaces were regenerated with (i) 10 mM NaOH and 500 mM NaCl or (ii) 3 M guanidinium hydrochloride (HC84.1 Fab–binding experiments). Kinetic analyses were performed in a running buffer containing 10 mM Hepes (pH 7.4), 150 mM NaCl, 3 mM EDTA, and 0.05% v/v Tween 20 with 1:2 dilution series for scaffolds and particles in duplicates. Blank injections were used for double referencing and a dimethyl sulfoxide calibration curve for bulk correction. Analysis and correction of the obtained data were performed using the Creoptix WAVE control software using a 1-to-1 binding model for kinetic analysis. A mass transport binding model was used for the IGH526 Fab–E1_S1 binding experiments.

### Quantification of capsid-like immunogen NPs in SDS-PAGE gel for immunization by densitometry analysis

The amount of the scaffold present in the sample of individual capsid-like immunogen NPs was quantified as follows. Flash-frozen aliquots of NPs adjusted to a concentration of 1 to 1.5 mg/ml were thawed to RT and spun at 18,213*g* for 10 min. Five microliters from the unboiled mix of capsid-like immunogen NPs (25 μl) with 5× SDS-PAGE sample buffer (5 μl) were loaded onto the SDS-PAGE well and subjected to SDS-PAGE electrophoresis. In-gel fluorescence imaging was performed with an excitation wavelength of Alexa Fluor 488 using Chemidoc MP to identify the monomer and homodimer_HBc_GFP bands. Densitometry analysis was performed on the Coomassie-stained gel using Image Lab Software from Bio-Rad to normalize the scaffold concentration for immunization in mice, i.e., 50 μl of the concentrate consisted of 15 to 25 μg of scaffold for the individual NPs and for the NP mix, 4 to 6 μg of each of the scaffold, which is equivalent to total protein content of 75 μg per dose. The quantities of scaffold were calculated by considering the molar mass percentage of an intact HBc_homodimer flanked with GFP molecules, one protomer depleted of GB1 and the other flanked with the respective scaffold.

### Negative stain electron microscopy of capsid-like immunogen NPs

Grids (Ultrathin carbon film on a copper support grid, 400 Mesh, Electron Microscopy Science) were plasma cleaned for 30 s with the PDC-32G-2 Basic Plasma Cleaner (Harrick Plasma). Five microliters of the sample at a concentration of 0.15 mg/ml in 25 μM tris (pH 7.5) and 500 mM NaCl was applied onto the grid and incubated for 2 min at RT. Then, the grids were washed three times with water and stained afterward with 2% (w/v) uranyl formate for 30 s. The grids were imaged on a Morgagni 268 microscope operated at 80 kV equipped with a Wolfram filament. Images were taken with a Side-Mount Veleta camera 2x2K (Olympus Soft Imaging Solutions, Münster).

### Immunization of C57BL6 and human antibody mice

Seventeen female C57BL6 mice (5 to 6 weeks old) or 10 human antibody mice were immunized each with ~9 μg of scaffold based on densitometric analyses shown in fig. S7 and the expected occupancy of the scaffold per HBc NP within 50 μl of individual CLPs (control – delGB1_HBc, NP Epi E1, or NP E2 EpiI) or 100 μl of combination of both scaffold capsid-like NPs (NP Epi E1 and NP E2 EpiI) or with 2 μg of soluble E2 ectodomain (genotype 2a-3). The latter dose was selected in view of two recent studies demonstrating an efficient antibody induction using doses of 1 to 2 μg (*87*) and 1 μg (*88*). Fresh NP aliquots were thawed on ice for each vaccination and spun at 18,213*g*, 10 min using a tabletop centrifuge at 4°. After centrifugation, the supernatant was transferred to a fresh tube and used for vaccination studies. Each animal was vaccinated four times at a 2-week interval (weeks 0, 2, 4, and 6), thereby allowing for a straightforward evaluation of prime-boost strategies by switching antigens between weeks 0 and 6. Antigens (NPs or soluble proteins) with cyclic-di–adenosine monophosphate (7.5 μg per dose) were mixed with equal volume of alum and injected either intraperitoneally (NPs) or intramuscularly (soluble protein). For the latter, for each animal, the inoculum was split into two equal doses, which were applied to the right and left thigh muscles. Preimmunization samples were collected at week 0 before the first injection. Terminal bleeds were performed at week 9. Blood samples were centrifuged at 4000*g*, and the serum collected was stored in aliquots at −80°C until use for ELISA, neutralization, and antibody purification assays. All procedures were performed according to European and German legislation and were approved by the local Animal Ethics Committee in Regensburg for C57BL6 mice (animal study approval numbers 54-2532.4-04/14 and 55.2.2-2532.2-953-20) and in Erlangen for human antibody mice, respectively (animal study approval number 55.2.2-2532.2-961).

### Flow cytometric sorting of B cells, sequencing, and analysis

Frozen splenocytes from four human antibody mice immunized with NPs and four mice immunized with recombinant HCV E2 were thawed and enriched for CD19^+^ cells using CD19 MicroBeads (mouse) and LS column (both Miltenyi Biotec). Fc receptors of the CD19^+^ splenocytes were blocked using rat anti-mouse CD16/CD32 (1/100). Next, the splenocytes were stained with rat anti-mouse CD8a (1/400), CD4 (1/400), F4/80 (1/100), and Ly6G/Ly-6C (Gr-1; 1/400) (all four antibodies were containing eFluor 450 fluorophore), CD83 (1/400, Alexa Fluor 700), CD45R (1/200, APC), IgM (1/200, PerCP-eFluor 710), and IgG1 (1/400, Brilliant Violet 750, BD Biosciences). All the antibodies except rat anti-mouse IgG1 were purchased from eBioscience. Last, the equimolar amounts of pHA4 sE2_GT2a_3, E1_S1, E2_S2_1 antigens fused to mRuby2 fluorescent protein (all 0.73 μM) and of E1_S1WT, E2_S2WT antigens fused to GFP (both 4.7 μM) were added to the staining mixture. DAPI (4′,6-diamidino-2-phenylindole) staining was used to identify dead cells. CD19^+^ splenocytes from both immunized mice groups exhibiting DAPI^−^, CD8a^−^, CD4^−^, F4/80^−^, Ly6G/Ly-6C^−^, IgM^−^, E1_S1WT_GFP^−^, E2_S2WT_GFP^−^, CD83^+^, CD45R^+^, IgG1^+^, pHA4_sE2_GT2a_3_mRuby2^+^, E1_S1_mRuby2^+^, and E2_S2_1_mRuby2^+^ phenotype were sorted at Central Research Facility Cell Sorting, Hannover Medical School on a BD Bioscience FACSAria III Fusion sorter (fig. S11). The sorted cell suspensions were processed with the Chromium Next GEM Single Cell V(D)J Reagent Kits v1.1. Sequencing of the scBCR libraries was performed on the Illumina NovaSeq 6000 PE150 platform. The flow cytometry data were analyzed using the FlowJo software, while the sequencing data were analyzed with the Loupe V(D)J Browser, and productive sequences were reannotated with IMGT/HighV-Quest.

### HCVcc neutralization assay

For inhibition of HCV infection, 200 μl of a Huh7.5 cell suspension was seeded into each well of a 96-well plate 24 hours before inoculation (10,000 cells per well). Luciferase reporter viruses of strains Gt1b_J4, Gt2a_Jc1, Gt3a_S52, and Gt5a_SA13 were mixed with indicated mouse sera (sera have been heat inactivated at 56°C for 30 min and used in a final dilution of 1:20) and preincubated for 45 min at 37°C. This mixture was used to inoculate cells for 4 hours in triplicates per serum. Thereafter, 170 μl of cell culture medium was added onto the cells. Viral infection was determined 72 hours after infection. Therefore, the supernatant was removed, and the cells were washed with PBS and subsequently lysed by addition of 35 μl of MilliQ water, one freeze/thaw cycle and resuspension of cells. After addition of luciferase substrate (1 μM colenterazine; P.J.K., Kleinbittersdorf, Germany; in PBS), relative light units were measured in a plate luminometer (Lumat LB Centro, Berthold, Germany).

### Enzyme-linked immunosorbent assay

Nunc-Immuno Maxisorp 96-well plates were coated with 50 μl of soluble E2 glycoproteins or scaffolds at 0.5 to 5 μg/ml concentration incubated overnight at 4°C. Ninety-six–well plates were then washed with 300 μl of 1× PBS supplemented with 0.05% Tween-20 (PBST) and blocked with 100 μl of 10% milk to minimize unspecific binding. The plates were then washed once with 1× PBST and further incubated for 1 hour at RT with 50 μl of mice sera or IgGs at appropriate dilutions or in w/v. Excess or unbound Abs were washed thrice again with 1× PBST and incubated with the 50 μl of secondary antibody anti-human or anti-mouse horseradish peroxidase for 1 hour at RT. Unbound 2° Ab was removed by washing thrice again with PBST before treating with a 100 μl mixture of equal volumes of substrates TMB A + TMB B. The plates were incubated for 10 min, and peroxidase reaction was stopped using 50 μl of 1 M H_3_PO_4_ solution. The plates were immediately measured at 450 nm using a 96-well plate spectrophotometer.

### Statistical analysis

We analyzed differences in neutralization efficiency as a function of immunization strategy, HCV strains, and experimental batch using linear mixed-effects models and followed up with planned comparisons. Crucially, we used linear-mixed effects models as they explicitly account for dependencies in the data, here, repeated measures per individual mouse across four different HCV genotypes (*57*). We quantified the proportion of total variance that is due to inter-mouse variability by the unadjusted intra-class correlation coefficient [ICC; (*89*)]. For consistency, neutralization efficiency was defined as % infectivity relative to the mean % infectivity of mice immunized with control NPs throughout. We applied a Box-Cox transformation (λ = 1.4) to raw % infectivity results of C57BL6 mice to satisfy linear model assumptions. For C57BL6 mice, we additionally analyzed neutralization efficiency by the ratio of individual neutralization responses in post- versus preimmune serum. Note that this post versus pre within-mouse comparison provides additional compelling evidence that is higher in statistical power than a between-mouse treatment versus control analysis. Unfortunately, preimmune sera were not available for the follow-up study on human antibody mice for which we therefore only report the established % infectivity metric of neutralization efficiency.

Linear mixed-effect models included immunization strategy, HCV genotype, as well as their interaction as fixed-effect predictors of interest, and experimental batch as nuisance regressor. Inter-mouse variability in neutralization response was accounted for by the inclusion of mice-specific random intercepts. We summarized the fixed-effect results by type III analysis of variance using Satterthwaite’s approximation of degrees of freedom and followed up on significant interactions of immunization strategy and HCV genotype by running simple linear models separately per HCV genotype. For the experiment on laboratory mice, we applied general linear tests for planned comparisons of NPmix with sE2, and the basic control group. We applied the Dunnett correction for multiple comparisons (*90*). For visualization, group-level neutralization results per immunization strategy and HCV strain were tested against a value of 100% infectivity using two-sided one-sample *t* tests. *P* values were corrected by controlling for the false discovery rate (*91*). Complete model results are reported in tables S3 and S4.

## References

[1] W. H. Organization, “Global Hepatitis Report 2021” (2021).

[2] L. Sandmann, B. Schulte, M. P. Manns, B. Maasoumy, Treatment of chronic hepatitis C: Efficacy, side effects and complications. Visc. Med. 35, 161–170 (2019). 31367613 10.1159/000500963 PMC6616049

[3] N. Scott, D. P. Wilson, A. J. Thompson, E. Barnes, M. El-Sayed, A. S. Benzaken, H. E. Drummer, M. E. Hellard, The case for a universal hepatitis C vaccine to achieve hepatitis C elimination. BMC Med. 17, 175 (2019). 31530275 10.1186/s12916-019-1411-9 PMC6749704

[4] J. K. Ball, A. W. Tarr, J. A. McKeating, The past, present and future of neutralizing antibodies for hepatitis C virus. Antiviral Res. 105, 100–111 (2014). 24583033 10.1016/j.antiviral.2014.02.013 PMC4034163

[5] R. Thimme, T cell immunity to hepatitis C virus: Lessons for a prophylactic vaccine. J. Hepatol. 74, 220–229 (2021). 33002569 10.1016/j.jhep.2020.09.022

[6] N. H. Shoukry, Hepatitis C vaccines, antibodies, and T cells. Front. Immunol. 9, 1480 (2018). 30002657 10.3389/fimmu.2018.01480 PMC6031729

[7] E. Scarselli, H. Ansuini, R. Cerino, R. M. Roccasecca, S. Acali, G. Filocamo, C. Traboni, A. Nicosia, R. Cortese, A. Vitelli, The human scavenger receptor class B type I is a novel candidate receptor for the hepatitis C virus. EMBO J. 21, 5017–5025 (2002). 12356718 10.1093/emboj/cdf529 PMC129051

[8] P. Pileri, Y. Uematsu, S. Campagnoli, G. Galli, F. Falugi, R. Petracca, A. J. Weiner, M. Houghton, D. Rosa, G. Grandi, S. Abrignani, Binding of hepatitis C virus to CD81. Science 282, 938–941 (1998). 9794763 10.1126/science.282.5390.938

[9] A. Kumar, R. A. Hossain, S. A. Yost, W. Bu, Y. Wang, A. D. Dearborn, A. Grakoui, J. I. Cohen, J. Marcotrigiano, Structural insights into hepatitis C virus receptor binding and entry. Nature 598, 521–525 (2021). 34526719 10.1038/s41586-021-03913-5 PMC8542614

[10] I. Benedicto, F. Molina-Jimenez, B. Bartosch, F. L. Cosset, D. Lavillette, J. Prieto, R. Moreno-Otero, A. Valenzuela-Fernandez, R. Aldabe, M. Lopez-Cabrera, P. L. Majano, The tight junction-associated protein occludin is required for a postbinding step in hepatitis C virus entry and infection. J. Virol. 83, 8012–8020 (2009). 19515778 10.1128/JVI.00038-09 PMC2715771

[11] A. Ploss, M. J. Evans, V. A. Gaysinskaya, M. Panis, H. You, Y. P. de Jong, C. M. Rice, Human occludin is a hepatitis C virus entry factor required for infection of mouse cells. Nature 457, 882–886 (2009). 19182773 10.1038/nature07684 PMC2762424

[12] K. Page, M. T. Melia, R. T. Veenhuis, M. Winter, K. E. Rousseau, G. Massaccesi, W. O. Osburn, M. Forman, E. Thomas, K. Thornton, K. Wagner, V. Vassilev, L. Lin, P. J. Lum, L. C. Giudice, E. Stein, A. Asher, S. Chang, R. Gorman, M. G. Ghany, T. J. Liang, M. R. Wierzbicki, E. Scarselli, A. Nicosia, A. Folgori, S. Capone, A. L. Cox, Randomized trial of a vaccine regimen to prevent chronic HCV infection. N. Engl. J. Med. 384, 541–549 (2021). 33567193 10.1056/NEJMoa2023345 PMC8367093

[13] S. E. Frey, M. Houghton, S. Coates, S. Abrignani, D. Chien, D. Rosa, P. Pileri, R. Ray, A. M. Di Bisceglie, P. Rinella, H. Hill, M. C. Wolff, V. Schultze, J. H. Han, B. Scharschmidt, R. B. Belshe, Safety and immunogenicity of HCV E1E2 vaccine adjuvanted with MF59 administered to healthy adults. Vaccine 28, 6367–6373 (2010). 20619382 10.1016/j.vaccine.2010.06.084 PMC2923449

[14] J. L. Law, C. Chen, J. Wong, D. Hockman, D. M. Santer, S. E. Frey, R. B. Belshe, T. Wakita, J. Bukh, C. T. Jones, C. M. Rice, S. Abrignani, D. L. Tyrrell, M. Houghton, A hepatitis C virus (HCV) vaccine comprising envelope glycoproteins gpE1/gpE2 derived from a single isolate elicits broad cross-genotype neutralizing antibodies in humans. PLOS ONE 8, e59776 (2013). 23527266 10.1371/journal.pone.0059776 PMC3602185

[15] T. Khera, P. Behrendt, D. Bankwitz, R. J. P. Brown, D. Todt, M. Doepke, A. G. Khan, K. Schulze, J. Law, M. Logan, D. Hockman, J. A. J. X. Wong, L. Dold, V. Gonzalez-Motos, U. Spengler, A. Viejo-Borbolla, L. J. Ströh, T. Krey, A. W. Tarr, E. Steinmann, M. P. Manns, F. Klein, C. A. Guzman, J. Marcotrigiano, M. Houghton, T. Pietschmann, Functional and immunogenic characterization of diverse HCV glycoprotein E2 variants. J. Hepatol. 70, 593–602 (2019). 30439392 10.1016/j.jhep.2018.11.003

[16] B. E. Correia, J. T. Bates, R. J. Loomis, G. Baneyx, C. Carrico, J. G. Jardine, P. Rupert, C. Correnti, O. Kalyuzhniy, V. Vittal, M. J. Connell, E. Stevens, A. Schroeter, M. Chen, S. Macpherson, A. M. Serra, Y. Adachi, M. A. Holmes, Y. Li, R. E. Klevit, B. S. Graham, R. T. Wyatt, D. Baker, R. K. Strong, J. E. Crowe Jr., P. R. Johnson, W. R. Schief, Proof of principle for epitope-focused vaccine design. Nature 507, 201–206 (2014). 24499818 10.1038/nature12966 PMC4260937

[17] F. Sesterhenn, C. Yang, J. Bonet, J. T. Cramer, X. Wen, Y. Wang, C. I. Chiang, L. A. Abriata, I. Kucharska, G. Castoro, S. S. Vollers, M. Galloux, E. Dheilly, S. Rosset, P. Corthesy, S. Georgeon, M. Villard, C. A. Richard, D. Descamps, T. Delgado, E. Oricchio, M. A. Rameix-Welti, V. Mas, S. Ervin, J. F. Eleouet, S. Riffault, J. T. Bates, J. P. Julien, Y. Li, T. Jardetzky, T. Krey, B. E. Correia, De novo protein design enables the precise induction of RSV-neutralizing antibodies. Science 368, eaay5051 (2020). 32409444 10.1126/science.aay5051 PMC7391827

[18] C. T. Schoeder, P. Gilchuk, A. K. Sangha, K. V. Ledwitch, D. C. Malherbe, X. Zhang, E. Binshtein, L. E. Williamson, C. E. Martina, J. Dong, E. Armstrong, R. Sutton, R. Nargi, J. Rodriguez, N. Kuzmina, B. Fiala, N. P. King, A. Bukreyev, J. E. Crowe, J. Meiler, Epitope-focused immunogen design based on the ebolavirus glycoprotein HR2-MPER region. PLOS Pathog. 18, e1010518 (2022). 35584193 10.1371/journal.ppat.1010518 PMC9170092

[19] K. Xu, P. Acharya, R. Kong, C. Cheng, G.-Y. Chuang, K. Liu, M. K. Louder, S. O’Dell, R. Rawi, M. Sastry, C.-H. Shen, B. Zhang, T. Zhou, M. Asokan, R. T. Bailer, M. Chambers, X. Chen, C. W. Choi, V. P. Dandey, N. A. Doria-Rose, A. Druz, E. T. Eng, S. K. Farney, K. E. Foulds, H. Geng, I. S. Georgiev, J. Gorman, K. R. Hill, A. J. Jafari, Y. D. Kwon, Y.-T. Lai, T. Lemmin, K. McKee, T. Y. Ohr, L. Ou, D. Peng, A. P. Rowshan, Z. Sheng, J.-P. Todd, Y. Tsybovsky, E. G. Viox, Y. Wang, H. Wei, Y. Yang, A. F. Zhou, R. Chen, L. Yang, D. G. Scorpio, A. B. McDermott, L. Shapiro, B. Carragher, C. S. Potter, J. R. Mascola, P. D. Kwong, Epitope-based vaccine design yields fusion peptide-directed antibodies that neutralize diverse strains of HIV-1. Nat. Med. 24, 857–867 (2018). 29867235 10.1038/s41591-018-0042-6 PMC6358635

[20] L. He, Y. Cheng, L. Kong, P. Azadnia, E. Giang, J. Kim, M. R. Wood, I. A. Wilson, M. Law, J. Zhu, Approaching rational epitope vaccine design for hepatitis C virus with meta-server and multivalent scaffolding. Sci. Rep. 5, 12501 (2015). 26238798 10.1038/srep12501 PMC4533164

[21] B. G. Pierce, E. N. Boucher, K. H. Piepenbrink, M. Ejemel, C. A. Rapp, W. D. Thomas Jr., E. J. Sundberg, Z. Weng, Y. Wang, Structure-based design of hepatitis C virus vaccines that elicit neutralizing antibody responses to a conserved epitope. J. Virol. 91, e01032-17 (2017). 28794021 10.1128/JVI.01032-17 PMC5625506

[22] B. G. Pierce, Z. Y. Keck, R. Wang, P. Lau, K. Garagusi, K. Elkholy, E. A. Toth, R. A. Urbanowicz, J. D. Guest, P. Agnihotri, M. C. Kerzic, A. Marin, A. K. Andrianov, J. K. Ball, R. A. Mariuzza, T. R. Fuerst, S. K. H. Foung, Structure-based design of hepatitis C Virus E2 glycoprotein improves serum binding and cross-neutralization. J. Virol. 94, e00704-20 (2020). 32878891 10.1128/JVI.00704-20 PMC7592221

[23] A. Sandomenico, A. Leonardi, R. Berisio, L. Sanguigno, G. Foca, A. Foca, A. Ruggiero, N. Doti, L. Muscariello, D. Barone, C. Farina, A. Owsianka, L. Vitagliano, A. H. Patel, M. Ruvo, Generation and characterization of monoclonal antibodies against a cyclic variant of hepatitis C virus e2 epitope 412-422. J. Virol. 90, 3745–3759 (2016). 26819303 10.1128/JVI.02397-15 PMC4794675

[24] D. X. Johansson, C. Voisset, A. W. Tarr, M. Aung, J. K. Ball, J. Dubuisson, M. A. A. Persson, Human combinatorial libraries yield rare antibodies that broadly neutralize hepatitis C virus. Proc. Natl. Acad. Sci. U.S.A. 104, 16269–16274 (2007). 17911260 10.1073/pnas.0705522104 PMC2042196

[25] N. Kato, H. Sekiya, Y. Ootsuyama, T. Nakazawa, M. Hijikata, S. Ohkoshi, K. Shimotohnol, Humoral immune response to hypervariable region 1 of the putative envelope glycoprotein (gp7O) of hepatitis C virus. J. Virol. 67, 3923–3930 (1993). 7685404 10.1128/jvi.67.7.3923-3930.1993 PMC237759

[26] Z.-Y. Keck, T.-K. Li, J. Xia, M. Gal-Tanamy, O. Olson, S. H. Li, A. H. Patel, J. K. Ball, S. M. Lemon, S. K. H. Foung, Definition of a conserved immunodominant domain on hepatitis C virus E2 glycoprotein by neutralizing human monoclonal antibodies. J. Virol. 82, 6061–6066 (2008). 18400849 10.1128/JVI.02475-07 PMC2395155

[27] J.-C. Meunier, R. S. Russell, V. Goossens, S. Priem, H. Walter, E. Depla, A. Union, K. N. Faulk, J. Bukh, S. U. Emerson, R. H. Purcell, Isolation and characterization of broadly neutralizing human monoclonal antibodies to the e1 glycoprotein of hepatitis c virus. J. Virol. 82, 966–973 (2008). 17977972 10.1128/JVI.01872-07 PMC2224608

[28] A. Owsianka, A. W. Tarr, V. S. Juttla, D. Lavillette, B. Bartosch, F.-L. Cosset, J. K. Ball, A. H. Patel, Monoclonal antibody AP33 defines a broadly neutralizing epitope on the hepatitis C virus E2 envelope glycoprotein. J. Virol. 79, 11095–11104 (2005). 16103160 10.1128/JVI.79.17.11095-11104.2005 PMC1193588

[29] M. Perotti, N. Mancini, R. A. Diotti, A. W. Tarr, J. K. Ball, A. Owsianka, R. Adair, A. H. Patel, M. Clementi, R. Burioni, Identification of a broadly cross-reacting and neutralizing human monoclonal antibody directed against the hepatitis C virus E2 protein. J. Virol. 82, 1047–1052 (2008). 17989176 10.1128/JVI.01986-07 PMC2224572

[30] G. Sautto, A. W. Tarr, N. Mancini, M. Clementi, Structural and antigenic definition of hepatitis C virus E2 glycoprotein epitopes targeted by monoclonal antibodies. Clin. Dev. Immunol. 2013, 450963 (2013). 23935648 10.1155/2013/450963 PMC3722892

[31] Y. K. Shimizu, S. M. Feinstone, M. Kohara, R. H. Purcell, H. Yoshikura, Hepatitis C virus: Detection of intracellular virus particles by electron microscop. Hepatology 23, 205–209 (1996). 8591842 10.1002/hep.510230202

[32] Y. Wang, Z.-Y. Keck, S. K. H. Foung, Neutralizing antibody response to hepatitis C virus. Viruses 3, 2127–2145 (2011). 22163337 10.3390/v3112127 PMC3230844

[33] E. Giang, M. Dorner, J. C. Prentoe, M. Dreux, M. J. Evans, J. Bukh, C. M. Rice, A. Ploss, D. R. Burton, M. Law, Human broadly neutralizing antibodies to the envelope glycoprotein complex of hepatitis C virus. Proc. Natl. Acad. Sci. U.S.A. 109, 6205–6210 (2012). 22492964 10.1073/pnas.1114927109 PMC3341081

[34] A. I. Flyak, S. Ruiz, M. D. Colbert, T. Luong, J. E. Crowe Jr., J. R. Bailey, P. J. Bjorkman, HCV broadly neutralizing antibodies use a CDRH3 disulfide motif to recognize an E2 glycoprotein site that can be targeted for vaccine design. Cell Host Microbe 24, 703–716.e3 (2018). 30439340 10.1016/j.chom.2018.10.009 PMC6258177

[35] A. I. Flyak, S. E. Ruiz, J. Salas, S. Rho, J. R. Bailey, P. J. Bjorkman, An ultralong CDRH2 in HCV neutralizing antibody demonstrates structural plasticity of antibodies against E2 glycoprotein. eLife 9, e53169 (2020). 32125272 10.7554/eLife.53169 PMC7064334

[36] L. Kong, E. Giang, T. Nieusma, R. U. Kadam, K. E. Cogburn, Y. Hua, X. Dai, R. L. Stanfield, D. R. Burton, A. B. Ward, I. A. Wilson, M. Law, Hepatitis C virus E2 envelope glycoprotein core structure. Science 342, 1090–1094 (2013). 24288331 10.1126/science.1243876 PMC3954638

[37] L. Kong, E. Giang, J. B. Robbins, R. L. Stanfield, D. R. Burton, I. A. Wilson, M. Law, Structural basis of hepatitis C virus neutralization by broadly neutralizing antibody HCV1. Proc. Natl. Acad. Sci. U.S.A. 109, 9499–9504 (2012). 22623528 10.1073/pnas.1202924109 PMC3386053

[38] L. Kong, R. U. Kadam, E. Giang, T. B. Ruwona, T. Nieusma, J. C. Culhane, R. L. Stanfield, P. E. Dawson, I. A. Wilson, M. Law, Structure of hepatitis C virus envelope glycoprotein E1 antigenic site 314-324 in complex with antibody IGH526. J. Mol. Biol. 427, 2617–2628 (2015). 26135247 10.1016/j.jmb.2015.06.012 PMC4523428

[39] T. Krey, A. Meola, Z. Y. Keck, L. Damier-Piolle, S. K. Foung, F. A. Rey, Structural basis of HCV neutralization by human monoclonal antibodies resistant to viral neutralization escape. PLOS Pathog. 9, e1003364 (2013). 23696737 10.1371/journal.ppat.1003364 PMC3656090

[40] A. Meola, A. W. Tarr, P. England, L. W. Meredith, C. P. McClure, S. K. Foung, J. A. McKeating, J. K. Ball, F. A. Rey, T. Krey, Structural flexibility of a conserved antigenic region in hepatitis C virus glycoprotein E2 recognized by broadly neutralizing antibodies. J. Virol. 89, 2170–2181 (2015). 25473061 10.1128/JVI.02190-14 PMC4338873

[41] J. A. Potter, A. M. Owsianka, N. Jeffery, D. J. Matthews, Z. Y. Keck, P. Lau, S. K. Foung, G. L. Taylor, A. H. Patel, Toward a hepatitis C virus vaccine: The structural basis of hepatitis C virus neutralization by AP33, a broadly neutralizing antibody. J. Virol. 86, 12923–12932 (2012). 22993159 10.1128/JVI.02052-12 PMC3497650

[42] A. Torrents de la Pena, K. Sliepen, L. Eshun-Wilson, M. L. Newby, J. D. Allen, I. Zon, S. Koekkoek, A. Chumbe, M. Crispin, J. Schinkel, G. C. Lander, R. W. Sanders, A. B. Ward, Structure of the hepatitis C virus E1E2 glycoprotein complex. Science 378, 263–269 (2022). 36264808 10.1126/science.abn9884 PMC10512783

[43] A. G. Khan, J. Whidby, M. T. Miller, H. Scarborough, A. V. Zatorski, A. Cygan, A. A. Price, S. A. Yost, C. D. Bohannon, J. Jacob, A. Grakoui, J. Marcotrigiano, Structure of the core ectodomain of the hepatitis C virus envelope glycoprotein 2. Nature 509, 381–384 (2014). 24553139 10.1038/nature13117 PMC4126800

[44] L. Kong, D. E. Lee, R. U. Kadam, T. Liu, E. Giang, T. Nieusma, F. Garces, N. Tzarum, V. L. Woods, A. B. Ward, S. Li, I. A. Wilson, M. Law, Structural flexibility at a major conserved antibody target on hepatitis C virus E2 antigen. Proc. Natl. Acad. Sci. U.S.A. 113, 12768–12773 (2016). 27791120 10.1073/pnas.1609780113 PMC5111675

[45] L. J. Stroh, K. Nagarathinam, T. Krey, Conformational flexibility in the CD81-binding site of the hepatitis C virus glycoprotein E2. Front. Immunol. 9, 1396 (2018). 29967619 10.3389/fimmu.2018.01396 PMC6015841

[46] Y. Li, B. G. Pierce, Q. Wang, Z. Y. Keck, T. R. Fuerst, S. K. Foung, R. A. Mariuzza, Structural basis for penetration of the glycan shield of hepatitis C virus E2 glycoprotein by a broadly neutralizing human antibody. J. Biol. Chem. 290, 10117–10125 (2015). 25737449 10.1074/jbc.M115.643528 PMC4400327

[47] A. W. Tarr, A. M. Owsianka, D. Jayaraj, R. J. P. Brown, T. P. Hickling, W. L. Irving, A. H. Patel, J. K. Ball, Determination of the human antibody response to the epitope defined by the hepatitis C virus-neutralizing monoclonal antibody AP33. J. Gen. Virol. 88, 2991–3001 (2007). 17947521 10.1099/vir.0.83065-0

[48] A. S. Peter, E. Roth, S. R. Schulz, K. Fraedrich, T. Steinmetz, D. Damm, M. Hauke, E. Richel, S. Mueller-Schmucker, K. Habenicht, V. Eberlein, L. Issmail, N. Uhlig, S. Dolles, E. Grüner, D. Peterhoff, S. Ciesek, M. Hoffmann, S. Pöhlmann, P. F. McKay, R. J. Shattock, R. Wölfel, E. Socher, R. Wagner, J. Eichler, H. Sticht, W. Schuh, F. Neipel, A. Ensser, D. Mielenz, M. Tenbusch, T. H. Winkler, T. Grunwald, K. Überla, H. M. Jäck, A pair of noncompeting neutralizing human monoclonal antibodies protecting from disease in a SARS-CoV-2 infection model. Eur. J. Immunol. 52, 770–783 (2022). 34355795 10.1002/eji.202149374 PMC8420377

[49] T. J. Broering, K. A. Garrity, N. K. Boatright, S. E. Sloan, F. Sandor, W. D. Thomas, G. Szabo, R. W. Finberg, D. M. Ambrosino, G. J. Babcock, Identification and characterization of broadly neutralizing human monoclonal antibodies directed against the E2 envelope glycoprotein of hepatitis C virus. J. Virol. 83, 12473–12482 (2009). 19759151 10.1128/JVI.01138-09 PMC2786766

[50] J. Gu, J. Hardy, I. Boo, P. Vietheer, K. McCaffrey, Y. Alhammad, A. Chopra, S. Gaudieri, P. Poumbourios, F. Coulibaly, H. E. Drummer, Escape of hepatitis C Virus from epitope I neutralization increases sensitivity of other neutralization epitopes. J. Virol. 92, e02066-17 (2018). 29467319 10.1128/JVI.02066-17 PMC5899191

[51] L. Kong, E. Giang, T. Nieusma, J. B. Robbins, M. C. Deller, R. L. Stanfield, I. A. Wilson, M. Law, Structure of hepatitis C virus envelope glycoprotein E2 antigenic site 412 to 423 in complex with antibody AP33. J. Virol. 86, 13085–13088 (2012). 22973046 10.1128/JVI.01939-12 PMC3497658

[52] H. Pantua, J. Diao, M. Ultsch, M. Hazen, M. Mathieu, K. McCutcheon, K. Takeda, S. Date, T. K. Cheung, Q. Phung, P. Hass, D. Arnott, J. A. Hongo, D. J. Matthews, A. Brown, A. H. Patel, R. F. Kelley, C. Eigenbrot, S. B. Kapadia, Glycan shifting on hepatitis C virus (HCV) E2 glycoprotein is a mechanism for escape from broadly neutralizing antibodies. J. Mol. Biol. 425, 1899–1914 (2013). 23458406 10.1016/j.jmb.2013.02.025

[53] J. López-Sagaseta, E. Malito, R. Rappuoli, M. J. Bottomley, Self-assembling protein nanoparticles in the design of vaccines. Comput. Struct. Biotechnol. J. 14, 58–68 (2016). 26862374 10.1016/j.csbj.2015.11.001 PMC4706605

[54] A. Walker, C. Skamel, M. Nassal, SplitCore: An exceptionally versatile viral nanoparticle for native whole protein display regardless of 3D structure. Sci. Rep. 1, 5 (2011). 22355524 10.1038/srep00005 PMC3216493

[55] J. Doerrbecker, M. Friesland, N. Riebesehl, C. Ginkel, P. Behrendt, R. J. Brown, S. Ciesek, H. Wedemeyer, C. Sarrazin, L. Kaderali, T. Pietschmann, E. Steinmann, Incorporation of primary patient-derived glycoproteins into authentic infectious hepatitis C virus particles. Hepatology 60, 508–520 (2014). 24771613 10.1002/hep.27190

[56] D. Bankwitz, A. Bahai, M. Labuhn, M. Doepke, C. Ginkel, T. Khera, D. Todt, L. J. Ströh, L. Dold, F. Klein, F. Klawonn, T. Krey, P. Behrendt, M. Cornberg, A. C. McHardy, T. Pietschmann, Hepatitis C reference viruses highlight potent antibody responses and diverse viral functional interactions with neutralising antibodies. Gut 70, 1734–1745 (2021). 33323394 10.1136/gutjnl-2020-321190 PMC8355883

[57] Z. Yu, M. Guindani, S. F. Grieco, L. Chen, T. C. Holmes, X. Xu, Beyond t test and ANOVA: Applications of mixed-effects models for more rigorous statistical analysis in neuroscience research. Neuron 110, 21–35 (2022). 34784504 10.1016/j.neuron.2021.10.030 PMC8763600

[58] D. Bankwitz, M. Doepke, K. Hueging, R. Weller, J. Bruening, P. Behrendt, J. Y. Lee, F. W. R. Vondran, M. P. Manns, R. Bartenschlager, T. Pietschmann, Maturation of secreted HCV particles by incorporation of secreted ApoE protects from antibodies by enhancing infectivity. J. Hepatol. 67, 480–489 (2017). 28438690 10.1016/j.jhep.2017.04.010

[59] M. A. Asensio, Y. W. Lim, N. Wayham, K. Stadtmiller, R. C. Edgar, J. Leong, R. Leong, R. A. Mizrahi, M. S. Adams, J. F. Simons, M. J. Spindler, D. S. Johnson, A. S. Adler, Antibody repertoire analysis of mouse immunization protocols using microfluidics and molecular genomics. MAbs 11, 870–883 (2019). 30898066 10.1080/19420862.2019.1583995 PMC6601537

[60] T. Weber, J. Potthoff, S. Bizu, M. Labuhn, L. Dold, T. Schoofs, M. Horning, M. S. Ercanoglu, C. Kreer, L. Gieselmann, K. Vanshylla, B. Langhans, H. Janicki, L. J. Stroh, E. Knops, D. Nierhoff, U. Spengler, R. Kaiser, P. J. Bjorkman, T. Krey, D. Bankwitz, N. Pfeifer, T. Pietschmann, A. I. Flyak, F. Klein, Analysis of antibodies from HCV elite neutralizers identifies genetic determinants of broad neutralization. Immunity 55, 341–354.e7 (2022). 34990590 10.1016/j.immuni.2021.12.003 PMC10089621

[61] L. Swadling, S. Capone, R. D. Antrobus, A. Brown, R. Richardson, E. W. Newell, J. Halliday, C. Kelly, D. Bowen, J. Fergusson, A. Kurioka, V. Ammendola, M. Del Sorbo, F. Grazioli, M. L. Esposito, L. Siani, C. Traboni, A. Hill, S. Colloca, M. Davis, A. Nicosia, R. Cortese, A. Folgori, P. Klenerman, E. Barnes, A human vaccine strategy based on chimpanzee adenoviral and MVA vectors that primes, boosts, and sustains functional HCV-specific T cell memory. Sci. Transl. Med. 6, 261ra153 (2014). 10.1126/scitranslmed.3009185 PMC4669853 25378645

[62] D. G. Bowen, C. M. Walker, Adaptive immune responses in acute and chronic hepatitis C virus infection. Nature 436, 946–952 (2005). 16107834 10.1038/nature04079

[63] S. B. Cashman, B. D. Marsden, L. B. Dustin, The humoral immune response to HCV: Understanding is key to vaccine development. Front. Immunol. 5, 550 (2014). 25426115 10.3389/fimmu.2014.00550 PMC4226226

[64] M. Sevvana, Z. Keck, S. K. Foung, R. J. Kuhn, Structural perspectives on HCV humoral immune evasion mechanisms. Curr. Opin. Virol. 49, 92–101 (2021). 34091143 10.1016/j.coviro.2021.05.002 PMC8319080

[65] V. J. Kinchen, G. Massaccesi, A. I. Flyak, M. C. Mankowski, M. D. Colbert, W. O. Osburn, S. C. Ray, A. L. Cox, J. E. Crowe, J. R. Bailey, Plasma deconvolution identifies broadly neutralizing antibodies associated with hepatitis C virus clearance. J. Clin. Invest. 129, 4786–4796 (2019). 31408439 10.1172/JCI130720 PMC6819096

[66] M. Law, T. Maruyama, J. Lewis, E. Giang, A. W. Tarr, Z. Stamataki, P. Gastaminza, F. V. Chisari, I. M. Jones, R. I. Fox, J. K. Ball, J. A. McKeating, N. M. Kneteman, D. R. Burton, Broadly neutralizing antibodies protect against hepatitis C virus quasispecies challenge. Nat. Med. 14, 25–27 (2008). 18064037 10.1038/nm1698

[67] J. Wang, S. Lisanza, D. Juergens, D. Tischer, J. L. Watson, K. M. Castro, R. Ragotte, A. Saragovi, L. F. Milles, M. Baek, I. Anishchenko, W. Yang, D. R. Hicks, M. Expòsit, T. Schlichthaerle, J. H. Chun, J. Dauparas, N. Bennett, B. I. M. Wicky, A. Muenks, F. DiMaio, B. Correia, S. Ovchinnikov, D. Baker, Scaffolding protein functional sites using deep learning. Science 377, 387–394 (2022). 35862514 10.1126/science.abn2100 PMC9621694

[68] T. Patra, K. Meyer, Y. Haga, E. K. Reagan, D. Weissman, R. Ray, Hepatitis C virus E1 and modified E2 delivered from an mRNA vaccine induces protective immunity. npj Vaccines 8, 42 (2023). 36934116 10.1038/s41541-023-00635-9 PMC10024013

[69] A. I. Mosa, D. S. Campo, Y. Khudyakov, M. G. AbouHaidar, A. J. Gehring, A. Zahoor, J. K. Ball, R. A. Urbanowicz, J. J. Feld, Polyvalent immunization elicits a synergistic broadly neutralizing immune response to hypervariable region 1 variants of hepatitis C virus. Proc. Natl. Acad. Sci. U.S.A. 120, e2220294120 (2023). 37276424 10.1073/pnas.2220294120 PMC10268328

[70] H. M. Berman, J. Westbrook, Z. Feng, G. Gilliland, T. N. Bhat, H. Weissig, I. N. Shindyalov, P. E. Bourne, The protein data bank. Nucleic Acids Res. 28, 235–242 (2000). 10592235 10.1093/nar/28.1.235 PMC102472

[71] A. Leaver-Fay, M. Tyka, S. M. Lewis, O. F. Lange, J. Thompson, R. Jacak, K. Kaufman, P. D. Renfrew, C. A. Smith, W. Sheffler, I. W. Davis, S. Cooper, A. Treuille, D. J. Mandell, F. Richter, Y. E. A. Ban, S. J. Fleishman, J. E. Corn, D. E. Kim, S. Lyskov, M. Berrondo, S. Mentzer, Z. Popović, J. J. Havranek, J. Karanicolas, R. Das, J. Meiler, T. Kortemme, J. J. Gray, B. Kuhlman, D. Baker, P. Bradley, ROSETTA3: An object-oriented software suite for the simulation and design of macromolecules. Methods Enzymol. 487, 545–574 (2011). 21187238 10.1016/B978-0-12-381270-4.00019-6 PMC4083816

[72] J. K. Leman, B. D. Weitzner, S. M. Lewis, J. Adolf-Bryfogle, N. Alam, R. F. Alford, M. Aprahamian, D. Baker, K. A. Barlow, P. Barth, B. Basanta, B. J. Bender, K. Blacklock, J. Bonet, S. E. Boyken, P. Bradley, C. Bystroff, P. Conway, S. Cooper, B. E. Correia, B. Coventry, R. Das, R. M. De Jong, F. DiMaio, L. Dsilva, R. Dunbrack, A. S. Ford, B. Frenz, D. Y. Fu, C. Geniesse, L. Goldschmidt, R. Gowthaman, J. J. Gray, D. Gront, S. Guffy, S. Horowitz, P. S. Huang, T. Huber, T. M. Jacobs, J. R. Jeliazkov, D. K. Johnson, K. Kappel, J. Karanicolas, H. Khakzad, K. R. Khar, S. D. Khare, F. Khatib, A. Khramushin, I. C. King, R. Kleffner, B. Koepnick, T. Kortemme, G. Kuenze, B. Kuhlman, D. Kuroda, J. W. Labonte, J. K. Lai, G. Lapidoth, A. Leaver-Fay, S. Lindert, T. Linsky, N. London, J. H. Lubin, S. Lyskov, J. Maguire, L. Malmström, E. Marcos, O. Marcu, N. A. Marze, J. Meiler, R. Moretti, V. K. Mulligan, S. Nerli, C. Norn, S. Ó’Conchúir, N. Ollikainen, S. Ovchinnikov, M. S. Pacella, X. Pan, H. Park, R. E. Pavlovicz, M. Pethe, B. G. Pierce, K. B. Pilla, B. Raveh, P. D. Renfrew, S. S. R. Burman, A. Rubenstein, M. F. Sauer, A. Scheck, W. Schief, O. Schueler-Furman, Y. Sedan, A. M. Sevy, N. G. Sgourakis, L. Shi, J. B. Siegel, D. A. Silva, S. Smith, Y. Song, A. Stein, M. Szegedy, F. D. Teets, S. B. Thyme, R. Y. R. Wang, A. Watkins, L. Zimmerman, R. Bonneau, Macromolecular modeling and design in Rosetta: Recent methods and frameworks. Nat. Methods 17, 665–680 (2020). 32483333 10.1038/s41592-020-0848-2 PMC7603796

[73] N. Ferguson, T. D. Sharpe, P. J. Schartau, S. Sato, M. D. Allen, C. M. Johnson, T. J. Rutherford, A. R. Fersht, Ultra-fast barrier-limited folding in the peripheral subunit-binding domain family. J. Mol. Biol. 353, 427–446 (2005). 16168437 10.1016/j.jmb.2005.08.031

[74] P. R. Dormitzer, Z. Y. Sun, G. Wagner, S. C. Harrison, The rhesus rotavirus VP4 sialic acid binding domain has a galectin fold with a novel carbohydrate binding site. EMBO J. 21, 885–897 (2002). 11867517 10.1093/emboj/21.5.885 PMC125907

[75] P. Rossi, S. S. T. Ramelot, R. Xiao, C. K. Ho, L. C. Ma, T. B. Acton, M. A. Kennedy, G. T. Montelione, 1H, 13C, and 15N resonance assignments for the protein coded by gene locus BB0938 of Bordetella bronchiseptica. J. Biomol. NMR 33, 197 (2005). 10.1007/s10858-005-2593-3 16331425

[76] L. A. Abriata, L. Banci, I. Bertini, S. Ciofi-Baffoni, P. Gkazonis, G. A. Spyroulias, A. J. Vila, S. Wang, Mechanism of Cu_A_ assembly. Nat. Chem. Biol. 4, 599–601 (2008). 18758441 10.1038/nchembio.110 PMC2596924

[77] D. Breitsprecher, E. Gherardi, W. M. Bleymüller, H. H. Niemann, Crystal structure of an engineered YopM-InlB hybrid protein. BMC Struct. Biol. 14, 1–9 (2014). 24669959 10.1186/1472-6807-14-12 PMC3986869

[78] D. A. Silva, B. E. Correia, E. Procko, Motif-driven design of protein-protein interfaces. Methods Mol. Biol 1414, 285–304 (2016). 27094298 10.1007/978-1-4939-3569-7_17

[79] A. Goldenzweig, M. Goldsmith, S. E. Hill, O. Gertman, P. Laurino, Y. Ashani, O. Dym, T. Unger, S. Albeck, J. Prilusky, R. L. Lieberman, A. Aharoni, I. Silman, J. L. Sussman, D. S. Tawfik, S. J. Fleishman, Automated structure- and sequence-based design of proteins for high bacterial expression and stability. Mol. Cell 63, 337–346 (2016). 27425410 10.1016/j.molcel.2016.06.012 PMC4961223

[80] S. F. Altschul, W. Gish, W. Miller, E. W. Myers, D. J. Lipman, Basic local alignment search tool. J. Mol. Biol. 215, 403–410 (1990). 2231712 10.1016/S0022-2836(05)80360-2

[81] R. C. Edgar, MUSCLE: Multiple sequence alignment with high accuracy and high throughput. Nucleic Acids Res. 32, 1792–1797 (2004). 15034147 10.1093/nar/gkh340 PMC390337

[82] S. F. Altschul, E. M. Gertz, R. Agarwala, A. A. Schäffer, Y. K. Yu, PSI-BLAST pseudocounts and the minimum description length principle. Nucleic Acids Res. 37, 815–824 (2009). 19088134 10.1093/nar/gkn981 PMC2647318

[83] B. Kuhlman, D. Baker, Native protein sequences are close to optimal for their structures. Proc. Natl. Acad. Sci. U.S.A. 97, 10383–10388 (2000). 10984534 10.1073/pnas.97.19.10383 PMC27033

[84] A. A. Gilmartin, B. Lamp, T. Rumenapf, M. A. Persson, F. A. Rey, T. Krey, High-level secretion of recombinant monomeric murine and human single-chain Fv antibodies from Drosophila S2 cells. Protein Eng. Des. Sel. 25, 59–66 (2012). 22160929 10.1093/protein/gzr058 PMC3258843

[85] M. Backovic, T. Krey, Stable Drosophila cell lines: An alternative approach to exogenous protein expression. Methods Mol. Biol. 1350, 349–358 (2016). 26820867 10.1007/978-1-4939-3043-2_17

[86] P. D. Adams, P. V. Afonine, G. Bunkóczi, V. B. Chen, I. W. Davis, N. Echols, J. J. Headd, L. W. Hung, G. J. Kapral, R. W. Grosse-Kunstleve, A. J. McCoy, N. W. Moriarty, R. Oeffner, R. J. Read, D. C. Richardson, J. S. Richardson, T. C. Terwilliger, P. H. Zwart, *PHENIX*: A comprehensive Python-based system for macromolecular structure solution. Acta Crystallogr. D Biol. Crystallogr. 66, 213–221 (2010). 20124702 10.1107/S0907444909052925 PMC2815670

[87] A. Landi, J. Law, D. Hockman, M. Logan, K. Crawford, C. Chen, J. Kundu, T. Ebensen, C. A. Guzman, L. Deschatelets, L. Krishnan, D. L. J. Tyrrell, M. Houghton, Superior immunogenicity of HCV envelope glycoproteins when adjuvanted with cyclic-di-AMP, a STING activator or archaeosomes. Vaccine 35, 6949–6956 (2017). 29089195 10.1016/j.vaccine.2017.10.072

[88] B. Akache, L. Deschatelets, B. A. Harrison, R. Dudani, F. C. Stark, Y. Jia, A. Landi, J. L. M. Law, M. Logan, D. Hockman, J. Kundu, D. L. Tyrrell, L. Krishnan, M. Houghton, M. J. McCluskie, Effect of different adjuvants on the longevity and strength of humoral and cellular immune responses to the HCV envelope glycoproteins. Vaccine 7, 204 (2019). 10.3390/vaccines7040204 PMC6963754 31816920

[89] S. Nakagawa, P. C. D. Johnson, H. Schielzeth, The coefficient of determination *R*^2^ and intra-class correlation coefficient from generalized linear mixed-effects models revisited and expanded. J. R. Soc. Interface 14, 20170213 (2017). 28904005 10.1098/rsif.2017.0213 PMC5636267

[90] P. H. Westfall, Multiple testing of general contrasts using logical constraints and correlations. J. Am. Stat. Assoc. 92, 299–306 (1997).

[91] Y. Benjamini, Y. Hochberg, Controlling the false discovery rate - a practical and powerful approach to multiple testing. J R Stat Soc B 57, 289–300 (1995).

