# Supplementary material for: Epitope-focused immunogens targeting the hepatitis C virus glycoproteins induce broadly neutralizing antibodies
Source: Sci Adv. 2024 Dec 6;10(49):eado2600. doi: 10.1126/sciadv.ado2600 (PMC11623273; doi:10.1126/sciadv.ado2600)
Supplement: Supplementary file 1 — Figs. S1 to S12 Tables S1 to S5 [file sciadv.ado2600_sm.pdf]

Science Advances
AAAS

Supplementary Materials for

Epitope-focused immunogens targeting the hepatitis C virus glycoproteins induce broadly neutralizing antibodies

Kumar Nagarathinam *et al.*

Corresponding author: Bruno E. Correia, bruno.correia@epfl.ch; Thomas Krey, thomas.krey@uni-luebeck.de

Sci. Adv. **10**, eado2600 (2024)
DOI: 10.1126/sciadv.ado2600

This PDF file includes:

Figs. S1 to S12
Tables S1 to S5

Fig. S1.

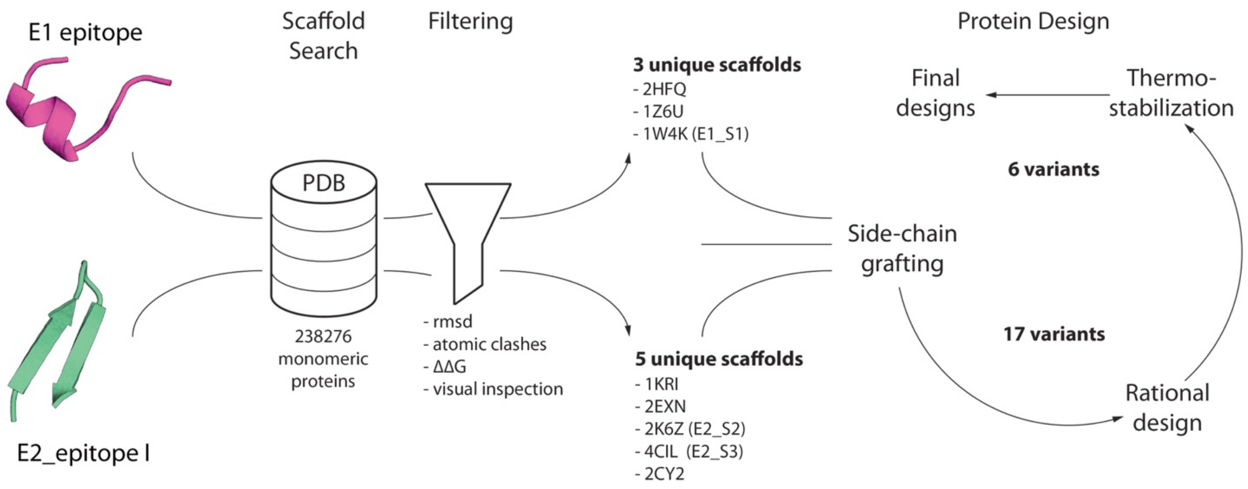

Workflow for computational design of scaffolds and thermostabilization of variants. Two conserved HCV neutralization epitopes, denoted as E1 epitope and E2 Epitope I, were extracted from crystal structures of antigens in complex with neutralizing antibody fragments IGH526 and HCV1 (PDB 4N0Y and 4DGV, respectively). A sub-database derived from the Protein Data Bank (PDB) containing 238,276 monomeric proteins was queried for close structural matches for each epitope, respectively. Identified scaffold proteins were subjected to multiple filtering steps, where scaffolds with high RMSD values, steric clashes, and predicted low binding affinities were excluded. Computational filtering was followed by visual inspection to highlight suitable scaffolds. For E1 epitope three scaffolds and for E2 Epitope I five scaffold candidates were identified. Sidechains of each epitope were grafted onto the selected scaffolds and subsequent structure-based, rational design was employed to improve epitope presentation and antibody binding. After high-throughput preliminary characterization, promising designs were subjected to an additional round of computational design for improved protein stability. In a final step, thermostabilized designs and their parent designs were biochemically and structurally characterized to identify lead immunogens.

Fig. S2.

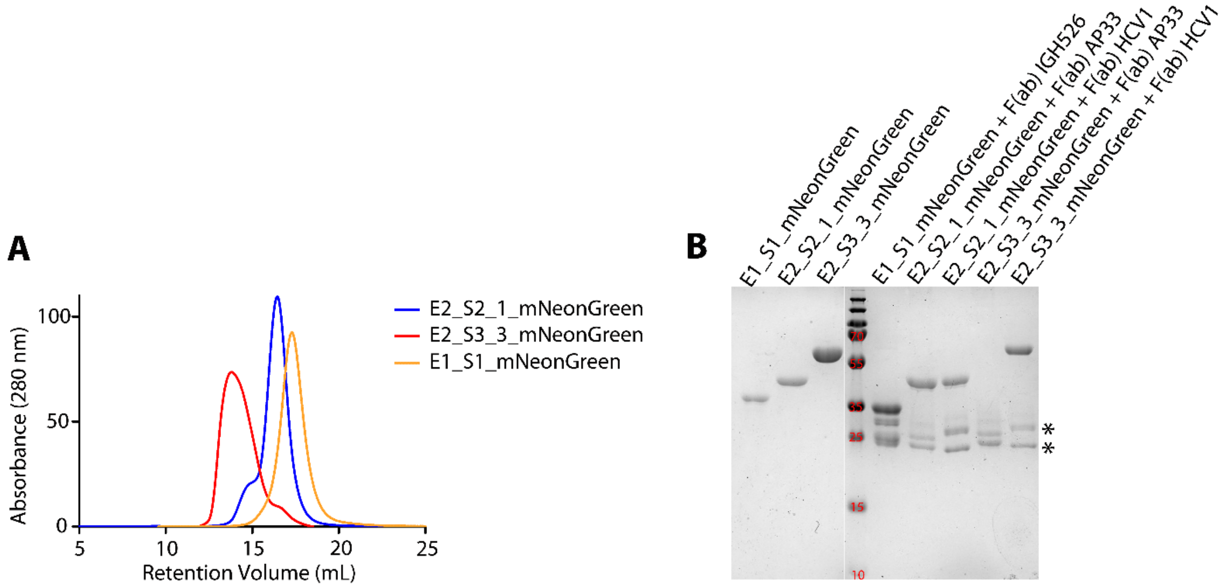

Preliminary characterization of immunogen lead candidates by pull-down assay and size-exclusion chromatographic profile of scaffold_mNeonGreen. a) Monodispersity of individual designs fused to mNeonGreen presenting E1 epitope (E1_S1) or E2 Epitope I (E2_S2_1, E2_S3_3) was assessed by size-exclusion chromatography using Superdex ® 200 Increase 10/300 GL column. b) Pull-down assay of individual designs fused to mNeonGreen using the respective cognate Fab fragments followed by SDS-PAGE analysis of the eluate and Coomassie staining reveals specific bands for heavy and light chain of the Fab indicated by an asterisk as well as for the individual designs if they bind to the respective Fab fragment. Of note, E2_S3_3 does not interact with AP33 Fab indicating the limited epitope accessibility on the design.

Fig. S3.

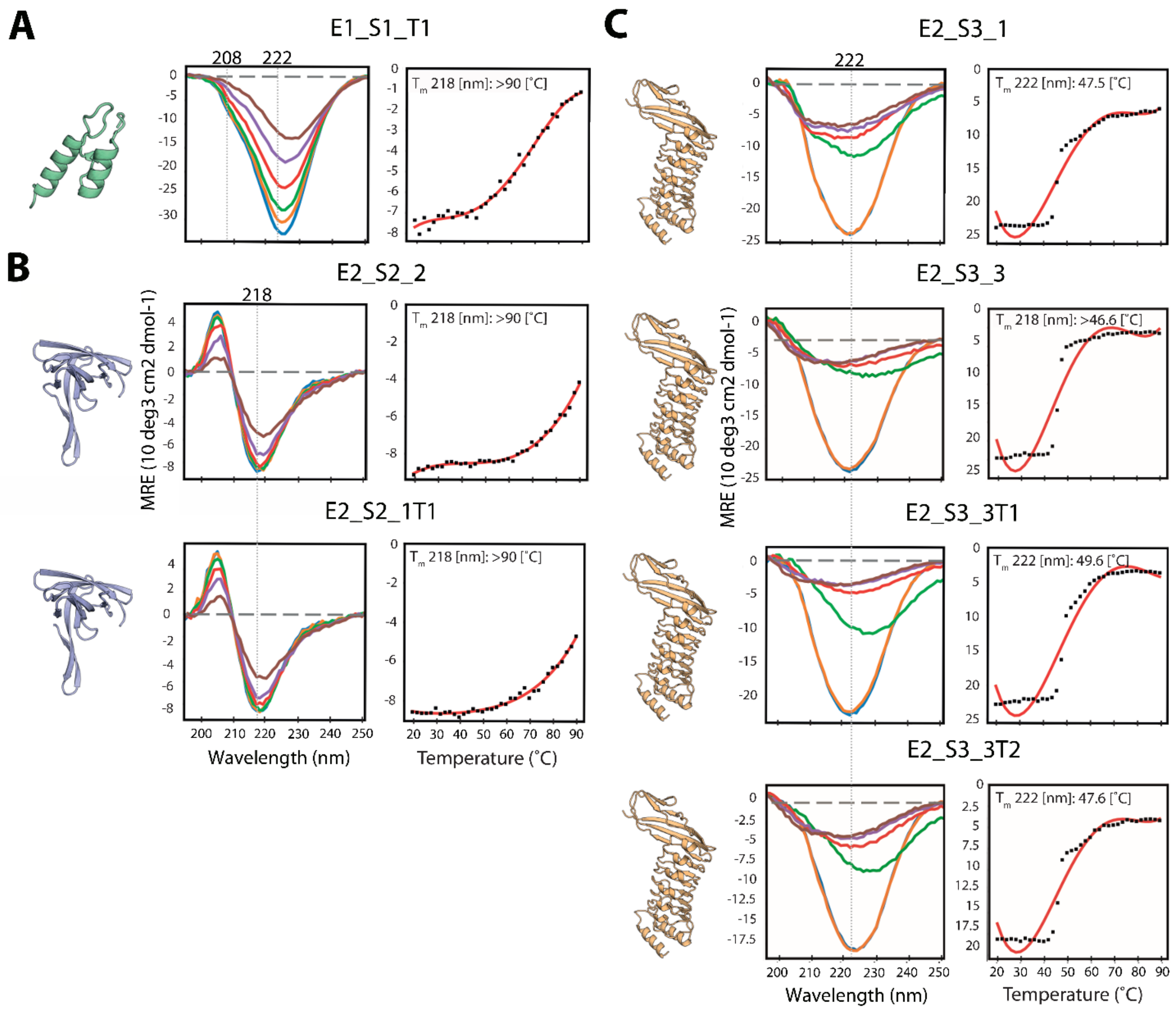

Secondary structure analysis and thermostability of designs. CD spectra reported as Mean Residual Ellipticity (MRE) were measured at different temperatures. The data points extracted from this ellipticity as a function of the increase in temperature from each of the CD spectra are indicated as black dots and fitted with a two-state model (red curve) that allows to derive the apparent CD-T_m from the slope. Typical positive maxima or negative minima of protein with α-helical or β-strand secondary structures are indicated with grey dotted lines. (A) Despite the high melting temperature, the observed CD spectrum for E1_S1_T1 does not show a negative minimum at 208 nm in contrast to the parent version, E1_S1 (see Figure 2). (B) CD spectra of E2_S2_2 and E2_S2_1T1 were very similar to the parent version, and E2_S2_1 displayed a high thermostability. (C) CD spectra of different E2_S3 versions indicated that these versions contained a mix of both α-helical and β-sheet secondary structures with minima only at 222 nm. The thermostabilized variants only showed a moderate increase in stability compared to the parent version from 47.5°C to 49.6°C.

Fig. S4.

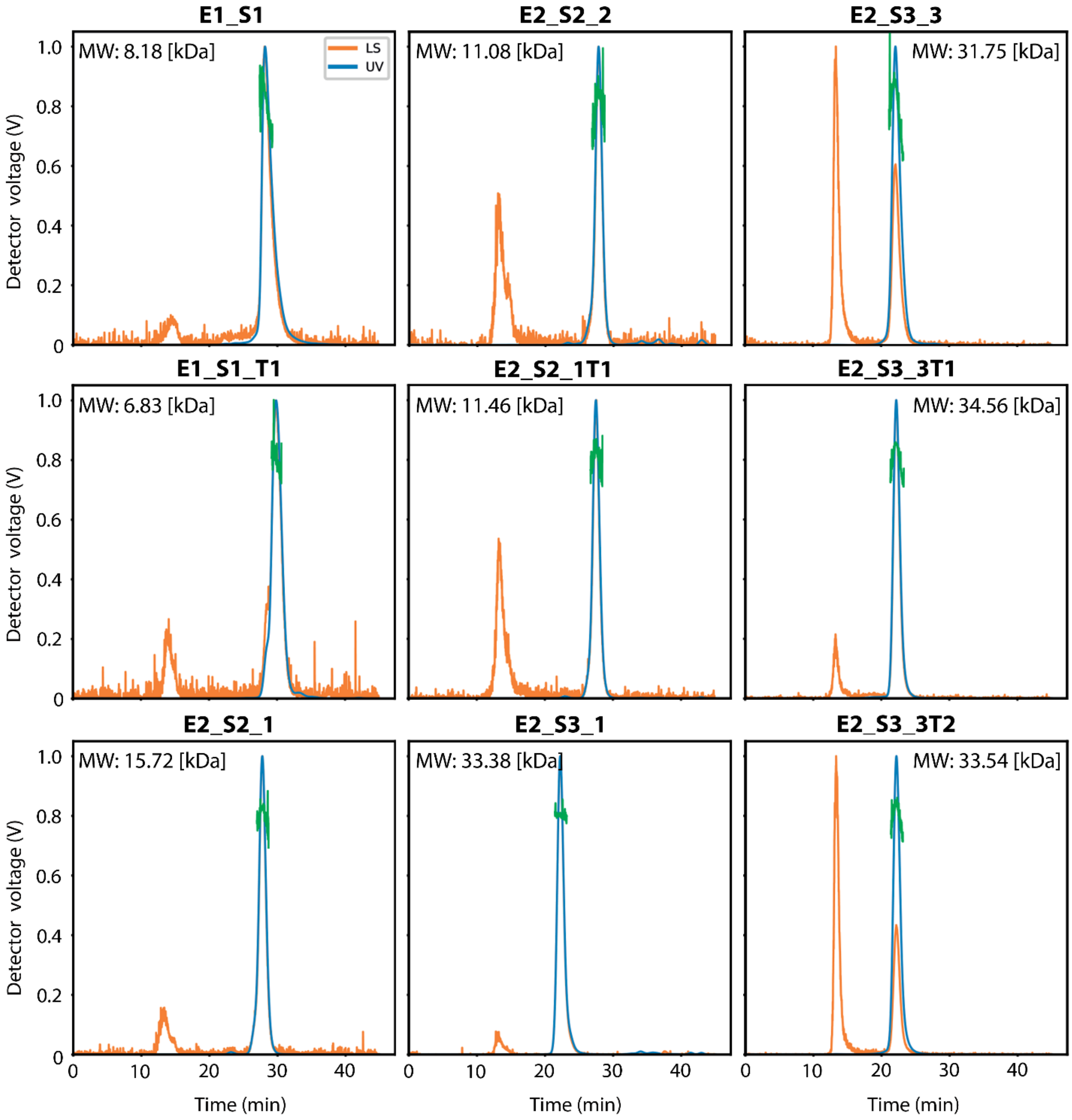

Size-exclusion chromatography and SEC-MALS profile of epitope designs. The chromatograms for each of the designs display UV absorption at 280 nm (blue) and the light scattering profile (orange). The curves together with the indicated molar mass of each peak were calculated by MALS (green).

Fig. S5.

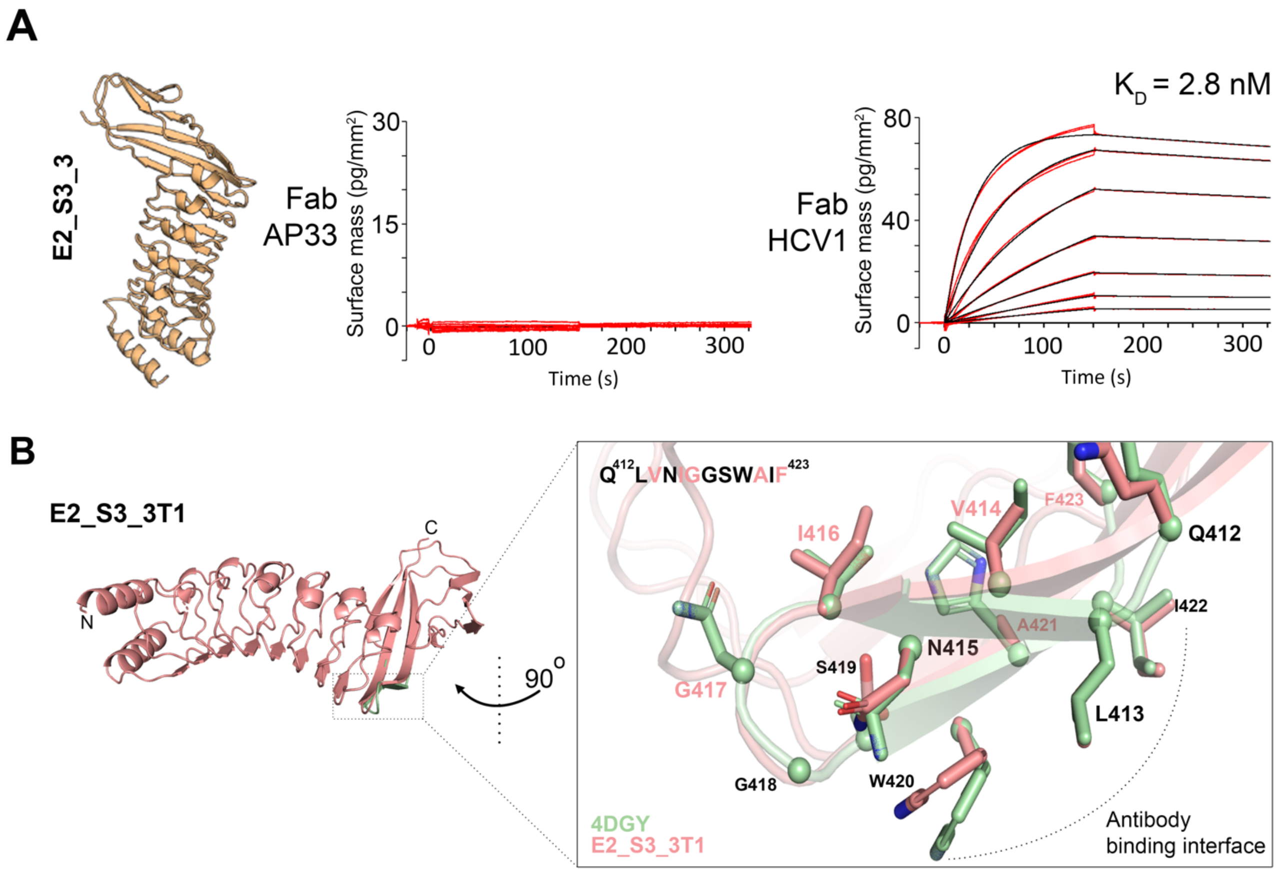

Characterization of E2_S3_3. (A) Sensorgrams derived from Grating-coupled interferometry (GCI)-measured binding kinetics for Fab-design interactions are shown with data in red and their respective fitting in black. The dissociation constant K_D for a 1-to-1 binding model or a mass transport binding model in case of E2_S3_3 – Fab HCV1 interaction is shown here and the kinetic parameters are shown in Table S5 (k_a, association rate constant; k_d, dissociation rate constant). Fab HCV1 binds with a dissociation constant K_D of 2.8 nM affinity, in contrast to Fab AP33, for which no binding was observed. (B) Superposition of the crystal structure of E2_S3_3T1 (right inset, salmon) onto the epitope of PDB model 4DGY (green), revealed a precise matching of the grafted epitope with the backbone conformation of the E2_epitope_I peptide (RMSD of Cα ≤ 0.5Å) and no differences were observed with the sidechain rotamers in the absence of the Fab demonstrating the accuracy of the design.

Fig. S6.

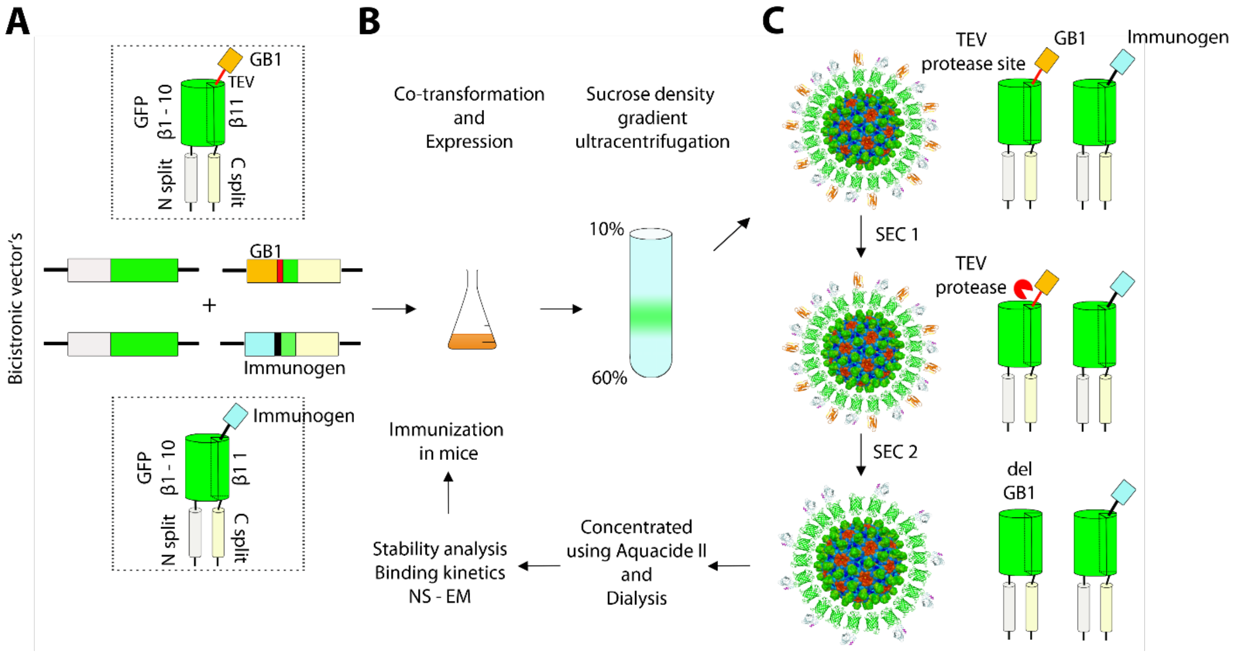

Production of capsid-like immunogen nanoparticles. (A) Two bicistronic vectors encoding for the N-split or C-split assembly subunit of the Hepatitis-B-virus capsid were co-transformed in BL21-CodonPlus (DE3)-RIPL *E.coli*. The N-split was fused with GFP lacking the eleventh β-strand of the β-barrel. This β-strand was fused to the C-split together with an extended linker to present either the design (cyan) or the B1 immunoglobulin-binding domain of protein G (GB1; orange) and a TEV protease cleavage site on the assembled capsid surface. (B, C) Icosahedral capsids displaying a mix of GB1 and immunogen at the surface were obtained from the bacterial cell lysate after sucrose density gradient ultracentrifugation. GB1 was proteolytically removed from the capsid using TEV protease giving rise to capsids presenting exclusively the design at the surface, albeit at lower occupancy. The capsid-like immunogen nanoparticles were concentrated using Aquacide II or alternatively using Amicon stirred cell concentrators, biophysically characterized extensively and then used for immunization.

Fig. S7.

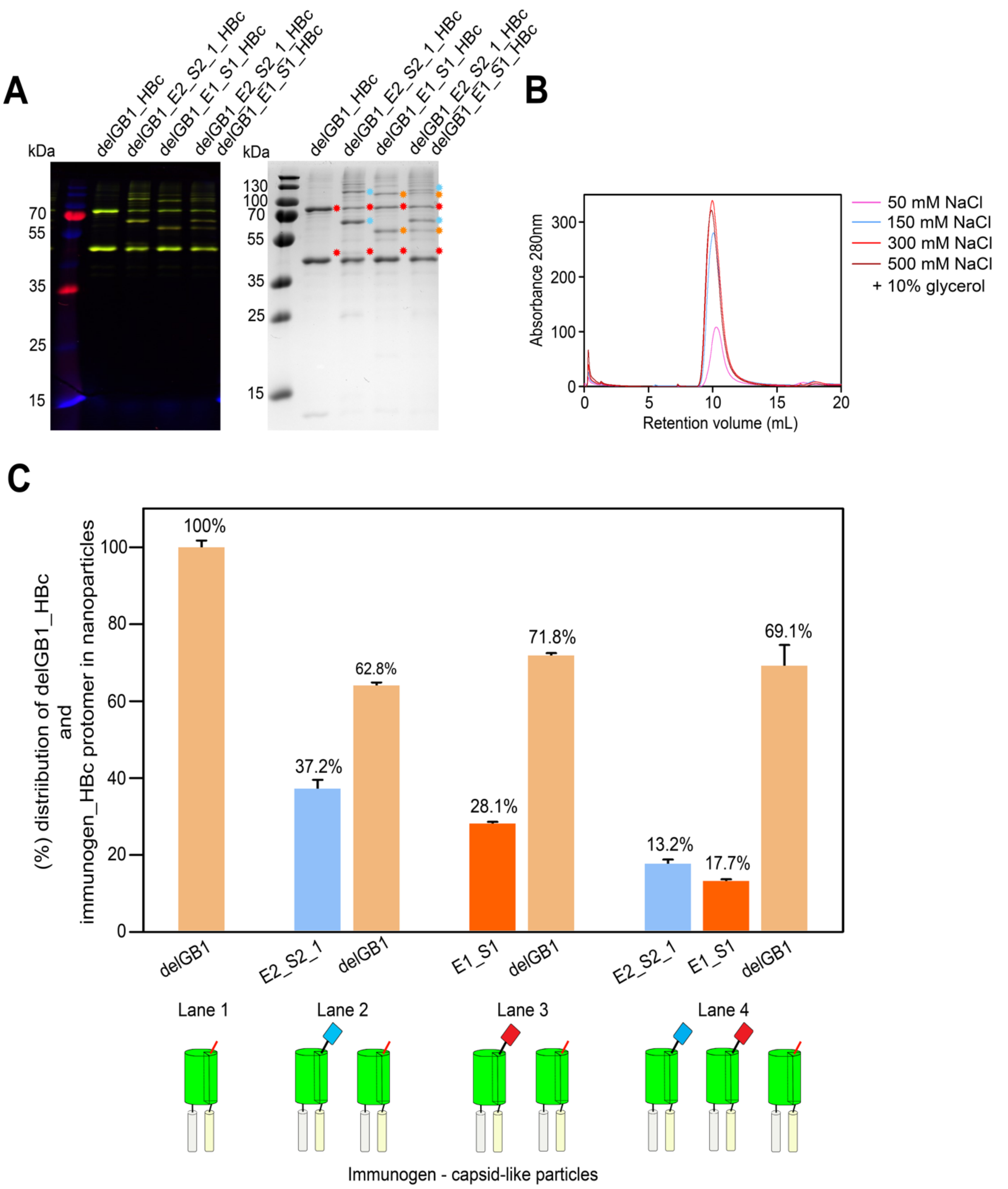

Densitometry analysis of capsid-like - immunogen nanoparticles in SDS-PAGE. (A) In-gel fluorescence and SDS-PAGE analysis followed by Coomassie staining of composite nanoparticles revealed an approximate occupancy of individual HBc assembly subunits (N- and C-split). HBc nanoparticles in the absence of epitope-scaffolds after proteolytical removal of GB1 were loaded as control (delGB1; lane 1). HBc_subunits run as monomers and as homodimers in each gel lane (red asterisks). Composite nanoparticles presenting also the epitope scaffolds run similarly as monomers and homodimers (E2_S2_1_HBc - cyan asterisks and E1_S1_HBc dark orange asterisks). Both composite nanoparticle populations were mixed and loaded for densitometry analysis in lane 4. (B)

Buffer optimization combined with the nanoparticle stability analysis was performed by SEC analysis of delGB1 capsids after 24h incubation at RT. (C) Bands corresponding to the delGB1_HBc or the respective immunogen_HBc nanoparticles shown in panel A were quantified using Image Lab software and the respective intensity distributions for the individual nanoparticle populations (combined monomer and homodimer) are shown in the diagram. Based on this occupancy approximation the total protein concentration was adjusted to achieve equivalent amounts of scaffold on the nanoparticles in µg/50 µl.

Fig. S8.

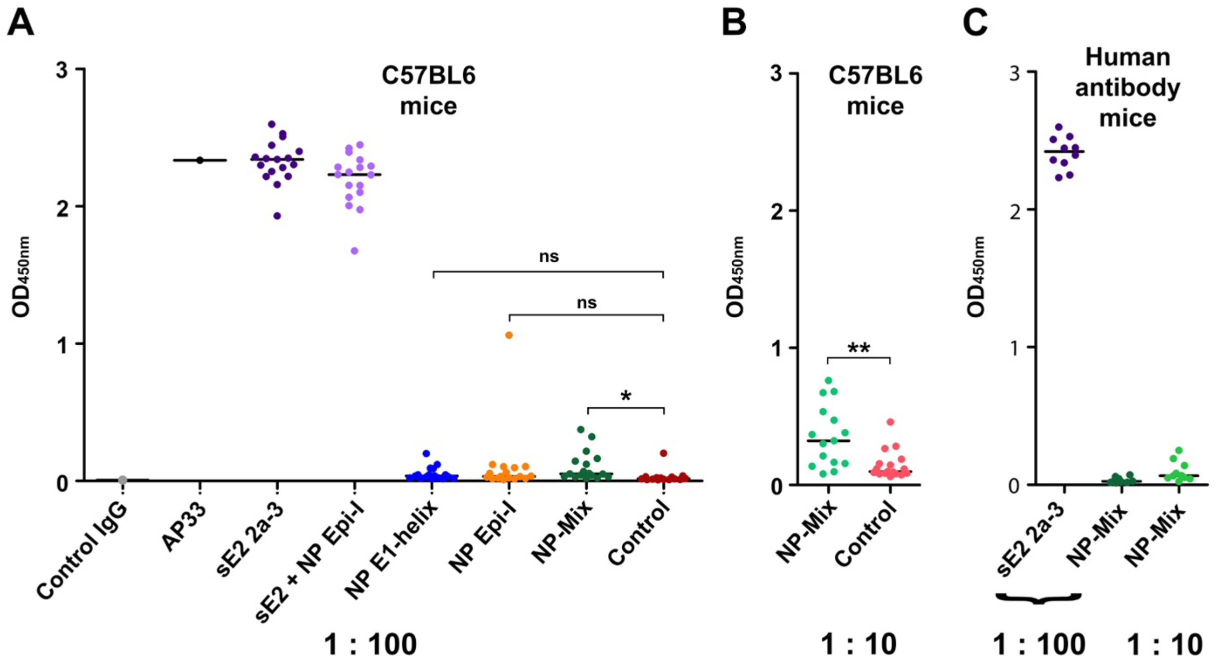

ELISA of six cohorts of female C57BL6 mice sera against sE2_GT_UKN2b_2.8. (A) An ELISA using a 1:100 sera dilution from each C57BL6 mice of the six cohorts (n=17) against sE2_GT_UKN2b_2.8 revealed that each cohort that was immunized at least once with sE2 developed high titers of binding antibodies recognizing sE2, whereas mice immunized exclusively with nanoparticles did not mount a considerable E2-specific antibody response. Murine AP33, which recognizes E2_EpiI and an unrelated isotype control were used as positive and negative control, respectively. (B) The same ELISA with a 1:10 sera dilution revealed a significant difference between mice immunized with NPmix and the control mice. An unpaired T test analysis was performed in comparison with the corresponding pre-immune serum. (C) An ELISA as shown in A+B was performed with sera from human antibody mice, demonstrating low overall E2-specific antibody levels in mice immunized exclusively with nanoparticles. ELISA results are presented as mean of three biological replicates with three technical replicates each. ns = $P > 0.05$; * = $P \leq 0.05$; ** = $P \leq 0.01$; *** = $P \leq 0.001$.

Fig. S9

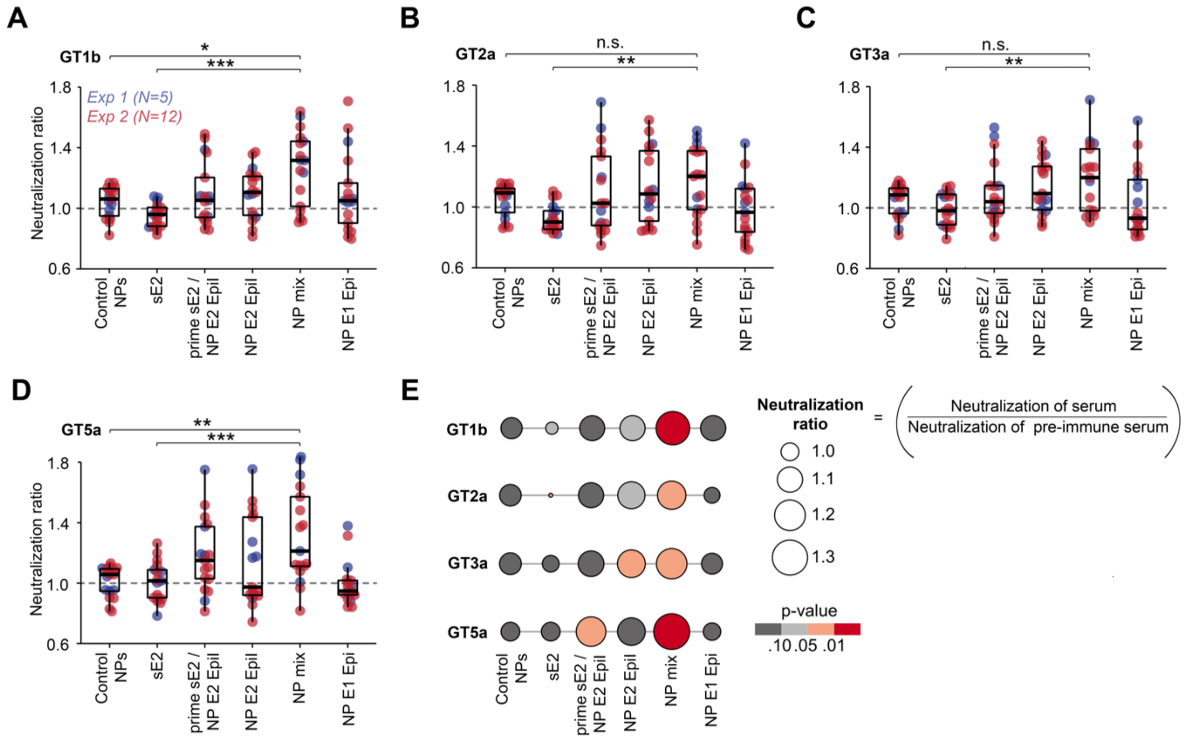

Nanoparticle mix induces potent bnAbs in C57BL6 mice. A single-dose neutralization assay was used to investigate the induction of nAbs against a panel of four different viruses that differ in neutralization sensitivity (Gt1b_J4, Gt2a_Jc1, Gt3a_S52 and Gt5a_SA13). (A to D). Female C57BL6 mice were immunized with the indicated immunogen(s) in two batches (N1=5 (blue), N2=12 (red)) and the neutralization efficiency of the individual mouse sera is reported as neutralization ratio (defined as neutralization of immune serum over neutralization of pre-immune serum) for the individual indicated viruses. Box plots show median center line, 25th to 75th percentile hinges, and whiskers indicate minimum and maximum within 1.5 × interquartile range. Neutralization efficiency was analyzed with linear mixed-effects regression indicating that inter-mice variability accounted for 52 % of total variance (intra-class correlation coefficient, ICC=0.52). Neutralization of NPmix was compared to sE2 and control by general linear tests with Dunnett-correction for multiple comparisons. To satisfy linear model assumption, raw neutralization ratios were inverse transformed for statistical analysis. (E) A bubble plot summarizes the neutralization outcome across the four HCVcc genotypes tested. For multiple comparisons, individual neutralization ratios were tested against 1 using two-sided t-tests with FDR-correction. N.s. = $P > 0.05$; * = $P \leq 0.05$; ** = $P \leq 0.01$; *** = $P \leq 0.001$.

Fig. S10

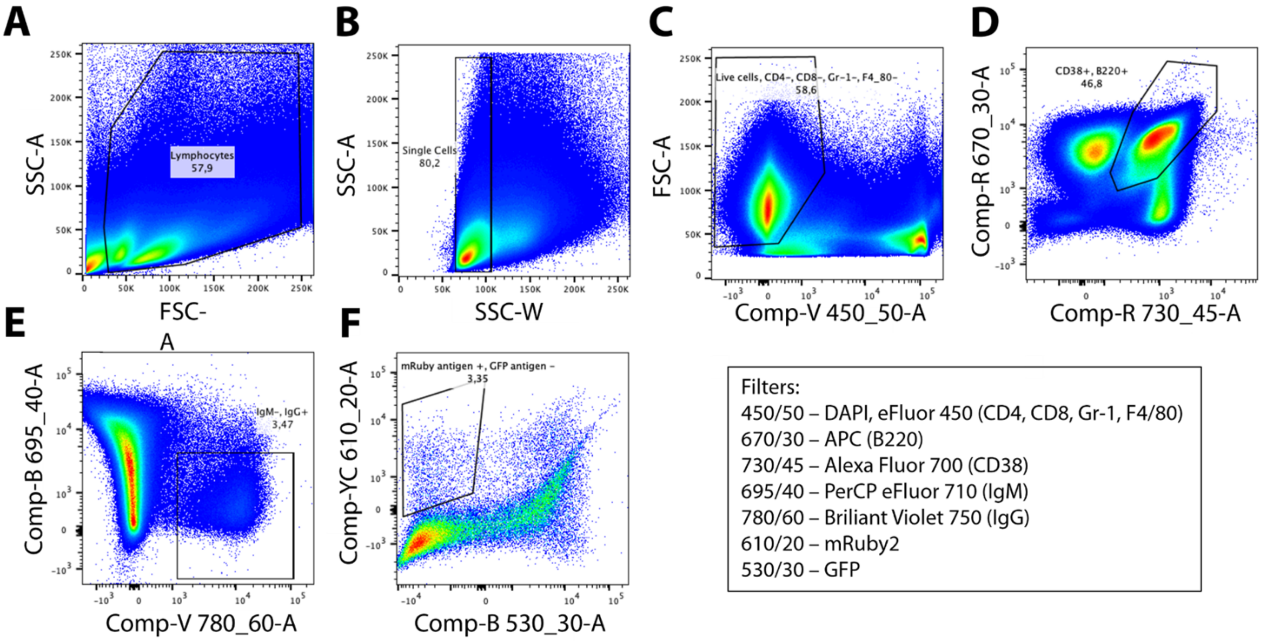

Flow cytometric sorting of mouse memory B cells recognizing HCV E1 and E2 glycoprotein epitopes. (A-F) Gating strategy for sorting of splenocytes from human antibody mice immunized with NPmix; lymphocytes were identified using forward (FSC-A) and side scatter (SSC-A; A) and single cells were selected by plotting width (SSC-W) vs. area (SSC-A) of the side scatter (B). Single lymphocytes were then gated for living (DAPI-), CD4-, CD8a-, Ly-6G/Ly-6C (Gr-1)- and F4/80- cells (C). Within these, CD38+ and CD45R B220+ population was identified (D) which was then used for selecting IgM- and IgG1+ cells (E). Finally, memory B cells binding to HCV E1/E2 epitopes were identified as those recognizing mRuby2 fused antigens (GT 2a-3 sE2, E1_S1 and E2_S2_1) and those not binding to wildtype antigens fused to GFP (E1_S1_WT, E2_S2_WT) (F). Data was analyzed using FlowJo.

Fig. S11

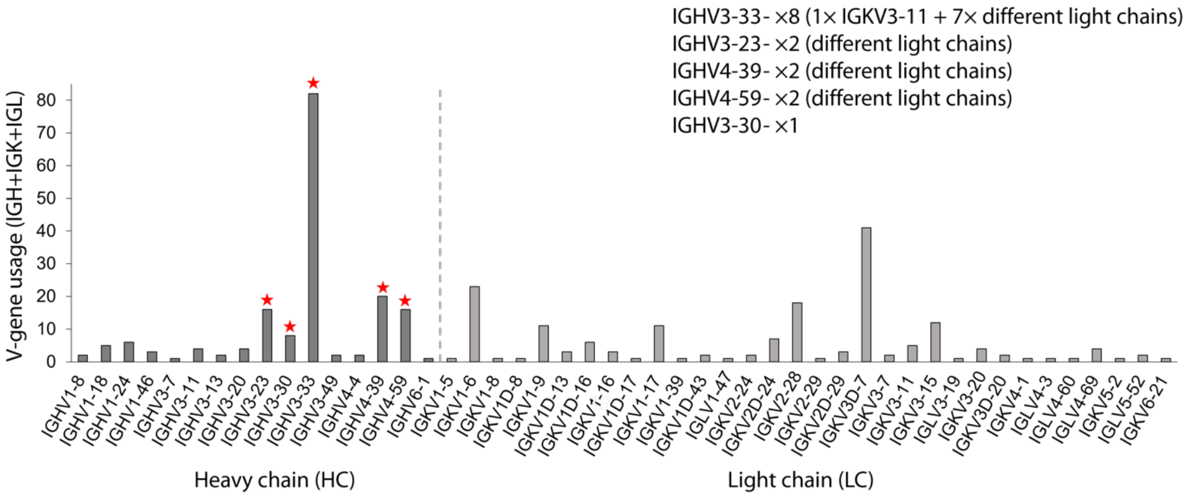

Selection of IgG clonotypes from human antibody mice immunized with NPmix. Single B-cell sequencing allowed evaluating the clonal frequency of germline V-gene usage of heavy chain (HC) and light chain (LC) of human antibody mice immunized with NPmix carrying E1_epitope and E2_EpitopeI. V-genes participating in productive IgG pairs that were selected based on clonotype and barcode frequency are highlighted by a red asterisk. The inset shows the number of IgGs selected for individual germline V-genes to evaluate their neutralization capacity against an HCVcc reference panel.

Fig. S12

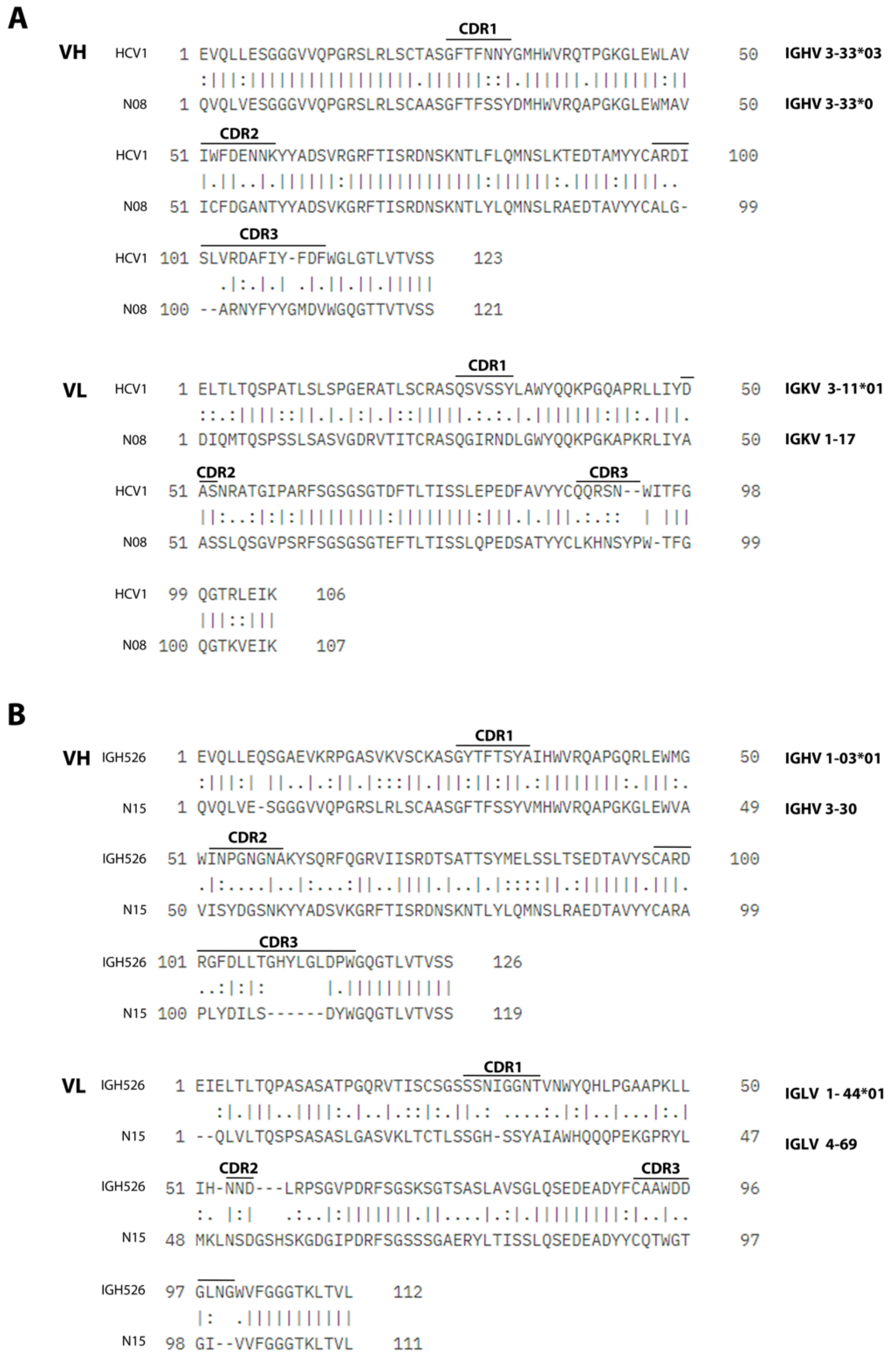

Sequence comparison of bnAbs isolated from human antibody mice with their parental antibodies used for scaffold design. Amino acid alignments of heavy (VH) and light chains (VL) of bnAbs N08 (A) and N15 (B) V-genes with the respective parental antibodies used for scaffold design HCV1 (A) and IGH526 (B). CDRs are indicated by black lines above the alignment and the germline V-gene shown on the right.

Table S1. X-ray data collection and refinement statistics of designed proteins in complex with their target antibodies or apo-form

	**1W4K_08 (E1_S1) + Fab IGH526**	**4CIL_04 (E2_S3_3T1)**
PDB code	8QP6	8QP7
Data collection	Proxima – 2A	Proxima – 2A
Wavelength	1.000677	1.0000
Resolution range	48.63 - 2.593 (2.686 - 2.593)	30.06 - 2.05 (2.123 – 2.05)
Space group	P 21 21 21	C121
Cell dimensions		
a, b, c (Å)	72.32, 121.76, 234.1	60.29, 31.67, 135.75
α, β, γ (°)	90.0, 90.0, 90.0	90, 94.314, 90
Total reflections	883486 (86769)	72172 (7544)
Unique reflections	64890 (6325)	16198 (1651)
Multiplicity	13.6 (13.7)	4.5 (4.6)
Completeness (%)	99.42 (97.58)	97.23 (95.02)
Mean I/sigma(I)	11.18 (0.74)	9.85 (2.54)
Wilson B-factor (Å^2)	70.17	30.83
Rmerge[a]	0.1644 (2.966)	0.1644 (1.088)
Refinement		
Reflections used in the refinement	64601 (6221)	16045 (1584)
Reflections used for R-free	3227 (309)	807 (80)
R-work[b]/R-free[c]	0.2320/0.2729	0.21445/0.2611
CC1/2[d]	0.997 (0.564)	0.98 (0.323)
No. Atoms	13212	2211
Protein	13205	2084
Solvent	7	127
Ion		
B-factors (Å^2)		
Average	77.91	39.75
Solvent	64.07	40.63
Ion		
R.m.s deviations		
Bond lengths (Å)	0.002	0.002
Bond angles (°)	0.58	0.48
Molprobity analysis		
Ramachandran favoured (%)	98.31	96.64
Ramachandran allowed (%)	1.69	3.36
Ramachandran outliers (%)	0.00	0.00
Rotamer outliers (%)	0.00	0.44
Clashscore	5.82	5.32

Values in parentheses indicate the statistics for the highest resolution shell.

[a]Rmerge = Σ|I − |/ΣI, where I is the integrated intensity of a given reflection and is the average intensity of multiple reflections.

[b]Rwork=Σ|Fobs − Fcal|/Σ|Fobs|, where Fobs and Fcal are the observed and calculated structural factors, respectively.

[c]Rfree was calculated as Rwork by using 5% of randomly chosen reflections omitted from the refinement. d CC1/2 refers to Pearson's correlation coefficients (CC) between intensity estimates from half data sets

Table S2. Amino acid sequences of characterized designs and their thermostable variants

Nomenclature	Scaffold designs	Amino acid sequences
E1_epitope		
E1_S1	1W4K_08	MGSREVAT^{314}PHRAAWLAMM^{324}LGIDASKVKGTGPGGVITVEDVKRWAEETAKATAGSENLYFQ
E1_S1_T1	1W4K_T1	MGSREVATPHRAAWLAMMLGIDASKVKGTGPGGVITVEDVKRAAEETAKATAGSENLYFQ
E2_Epitope_I		
E2_S2_1	2K6Z_01	MGSFTEGWVRFSPGPNAAAYLTLENPGDLPLRLVGARTPVAERVELWETQLINTNGSWHIGSRPVPFLEVPPKGRVELSPGGYYFHLLGLKRPLKAGEEVELDLLFAGGKVLKVVLPVEARGSENLYFQ
E2_S2_2	2K6Z_02	MGSFTEGWVRFSPGPNAAAYLTLENPGDLPLRLVGARTPVAERVELWETQLRNTNGSWHIGSRPVPFLEVPPKGRVELSPGGYYFHLLGLKRPLKAGEEVELDLLFAGGKVLKVVLPVEARENLYFQ
E2_S2_1T1	2K6Z_T1	MGSFTSGWVRFSPGPNAAAYLTLENPGDEPLRLVGARTPVAERVELWETQLINTNGSWHIGSRPVPFLEVPPKGRVELSPGGYYFHLLGLKRPLKAGEEVELDLLFEGGKVLKVVLPVEARGSENLYFQ
E2_S3_1	4CIL_01	MGAMGKSKAEYYNAWSEWERNAPPGEQRGMAVSRLRDALDRQAHELELNNLGLSSLPGIQYLPNVTKLFLNGNKLTDIKPLANLKNLGWLFLDENKVKDLSSLKDLKKLKSLSLEHNGISDINGLVHLPQLESLYLGNNKITDITVLSRLTKLDTLSLEDNQISDIVPLAGLTKLQNLYLSKNHISDLRALAGLKNLDVLELFSQEILNKPINHQSNLVVPNTVKDTDGSLTTPAIISDDGDYEKPNVKWHLPEFTNEVSFIFRQLVNIGGSWAIFSGRVTQPLKEGSENLYFQ
E2_S3_3	4CIL_03	MGKSKAEYYNAWSEWERNAPPGNGEQRGMAVSRLRDALDRQAHELELNNLGLSSLPGIQYLPNVTKLFLNGNKLTDIKPLANLKNLGWLFLDENKVKDLSSLKDLKKLKSLSLEHNGISDINGLVHLPQLESLYLGNNKITDITVLSRLTKLDTLSLEDNQISDIVPLAGLTKLQNLYLSKNHISDLRALAGLKNLDVLELFSQEILNKPINHQSNLVVPNTVKDTDGSLTTPAIISDDGDYEKPNVKWHLPEFTNEVSFIFRQLVNIAGSWAIFSGRVTQPLKEGSENLYFQ
E2_S3_3T1	4CIL_04	MGAMGKSKAEYYNAWSEWERNAPGGNGEQRGMAVSRLRDALDRQSHELELNNLGLSSLPGIQYLPNVTKLFLNGNKLTDIKPLANLKNLGWLFLDENKVKDLSSLKDLKKLKSLSLEHNGISDINGLVHLPQLESLYLGNNKITDITVLSRLTKLDTLSLEDNQISDIVPLAGLTKLQNLYLSKNHISDLRALAGLKNLDVLELFSQEILNKPINHQSNLVVPNTVKDTDGSLTTPAIISDDGDYEKPNVKWHLPEFTNEVSFIFRQLVNIGGSWAIFSGRVTQPLKEGSENLYFQ
E2_S3_3T2	4CIL_05	MGAMGKSKAEYYNAWSEWERNAPPGEQRGMAVSRLRDALDRQAHELELNNLGLSSLPGIQYLPNVTKLFLNGNKLTDIKPLANLKNLGWLFLDENKVKDLSSLKDLKKLKSLSLEHNGISDINGLVHLPQLESLYLGNNKITDITVLSRLTKLDTLSLEDNQISDIVPLAGLTKLQNLYLSKNHISDLRALAGLKNLDVLELFSQEILNKPINHQSNLVVPNTVKDTDGSLTTPAIISDDGDYEKPNVKWHLPEFTNEVSFIFRQLINTGGSWAIFSGRVTQPLKEGSENLYFQ

Table S3. Predicting % infectivity rel. to control in Experiment 1 (modelled using a Box-Cox transform with λ= 1.4)

**Analysis of variance of fixed effects**						
Predictors	*Sum Sq*	*Mean Sq*	*NumDF*	*DenDF*	*F*	*p*
HCV genotype	33885	11295	3	288	1.30	0.276
Immunogen	192641	38528	5	95	4.42	0.001
Batch	66021	66021	1	95	7.57	0.007
HCV genotype:immunogen	258352	17223	15	288	1.98	0.017
**Planned pairwise comparisons per genotype**						
	Estimate	*Std. Err.*	*t*	*p (adj.)*		
GT1b: NP mix vs. Control NPs	–148.67	59.08	–2.52	0.025		
GT1b: NP mix vs. sE2	–229.52	59.08	–3.89	0.0004		
GT2a: NP mix vs. Control NPs	–170.69	62.32	–2.74	0.014		
GT2a: NP mix vs. sE2	–183.66	62.32	–2.95	0.008		
GT3a: NP mix vs. Control NPs	–146.15	53.31	–2.74	0.014		
GT3a: NP mix vs. sE2	–168.73	53.31	–3.17	0.004		
GT5a: NP mix vs. Control NPs	–156.20	44.40	–3.52	0.001		
GT5a: NP mix vs. sE2	–97.20	44.40	–2.19	0.057		
**Random effects**						
σ^2	8719.59					
$\tau_{00\ mouse}$	17391.22					
ICC adjusted / unadjusted	0.67 / 0.54					
N_{mouse}	102					
Observations	408					
R^2 marginal / conditional	0.20 / 0.73					

Table S4. Predicting % infectivity rel. to control in Experiment 2

**Analysis of variance of fixed effects**						
Predictors	*Sum Sq*	*Mean Sq*	*NumDF*	*DenDF*	*F*	*p*
HCV genotype	166.0	55.3	3	51	0.13	0.943
Immunogen	4511.2	4511.2	1	16	10.41	0.005
Batch	31.2	31.2	1	16	0.07	0.792
HCV genotype:immunogen	166.0	55.3	3	51	0.13	0.943
**Pairwise comparisons per genotype**						
	Estimate	*Std. Err.*	*t*	*p (adj.)*		
GT1b: NP mix vs. sE2	–42.54	16.22	–2.62	0.019		
GT2a: NP mix vs. sE2	–35.36	13.70	–2.58	0.020		
GT3a: NP mix vs. sE2	–38.04	14.09	–2.70	0.016		
GT5a: NP mix vs. sE2	–35.73	13.86	–2.58	0.020		
**Random effects**						
σ^2	433.32					
$\tau_{00\ mouse}$	543.87					
ICC adjusted / unadjusted	0.56 / 0.40					
N_{mouse}	19					
Observations	76					
R^2 marginal / conditional	0.28 / 0.68					

Table S5. Binding Kinetics data of scaffold designs against cognate F(ab) fragment characterized using Creoptix

F(ab)	Scaffold design	R_{max} (pg/mm^2)	k_{on} (M^{-1}s^{-1})	k_{off} (s^{-1})	K_D (nM)
E1_epitope					
IGH526	E1_S1	16.040	1.32×10^6	1.81×10^{-3}	1.4
E2_Epitope_I					
AP33	E2_S2_1	23.256	1.49×10^5	6.68×10^{-3}	44.9
AP33	E2_S3_3	-	-	-	-
AP33	sE2	28.257	8.32×10^4	4.86×10^{-3}	58.4
HCV1	E2_S2_1	45.484	5.27×10^4	1.21×10^{-3}	23
HCV1	E2_S3_3	74.642	1.33×10^5	3.69×10^{-4}	2.8
HCV1	sE2	29.737	1.48×10^4	4.35×10^{-3}	294
